# Supplementary material for: Improving cardiovascular disease risk communication in NHS Health Checks: a qualitative study
Source: BMJ Open. 2019 Sep 3;9(8):e026058. doi: 10.1136/bmjopen-2018-026058 (PMC6731822; doi:10.1136/bmjopen-2018-026058)
Supplement: Supplementary data [file bmjopen-2018-026058supp001.pdf]

NHS

NHS Health Check Results Card

Patient Name	[patient name]
Date of NHS Health Check	[date of health check]
NHS Number	[NHS number]

Cardiovascular Risk Report

What's your heart age?

Your age is: [XX]

Your current heart age is about: [XX]

What's your risk of having a heart attack or stroke in the next 10 years?

Your risk score is [XX] %. This means:

☐ 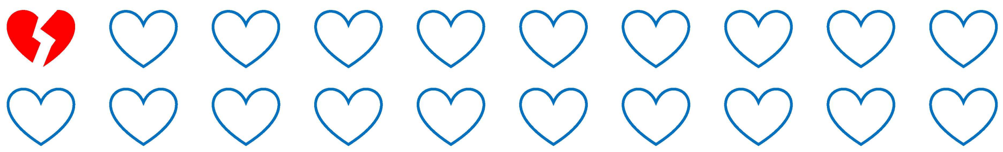

Low Risk

Between 1 and 2 of every 20 people like you will have a stroke or heart attack in the next 10 years

☐ 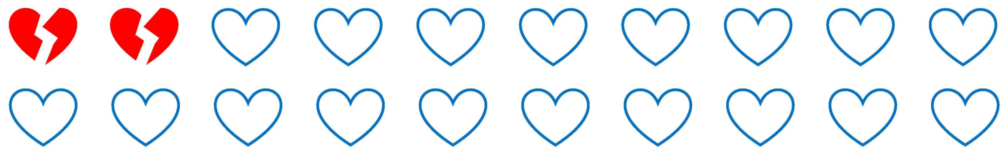

Medium Risk

Between 2 and 4 of every 20 people like you will have a stroke or heart attack in the next 10 years

☐ 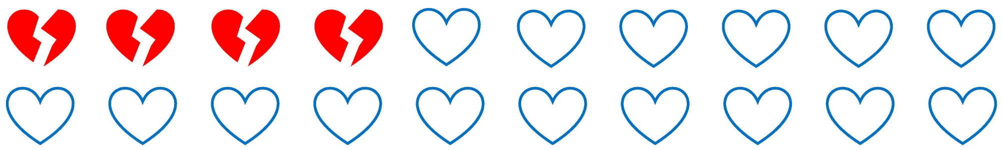

High Risk

More than 4 out of every 20 people like you will have a stroke or heart attack in the next 10 years

NHS HEALTH CHECK

Helping you prevent diabetes, heart disease, kidney disease, stroke & dementia

Developed by Queen Mary University of London, UCLPartners Academic Health Science Partnership

In partnership with NHS Newham Clinical Commissioning Group, Newham London

Every year in the UK.....

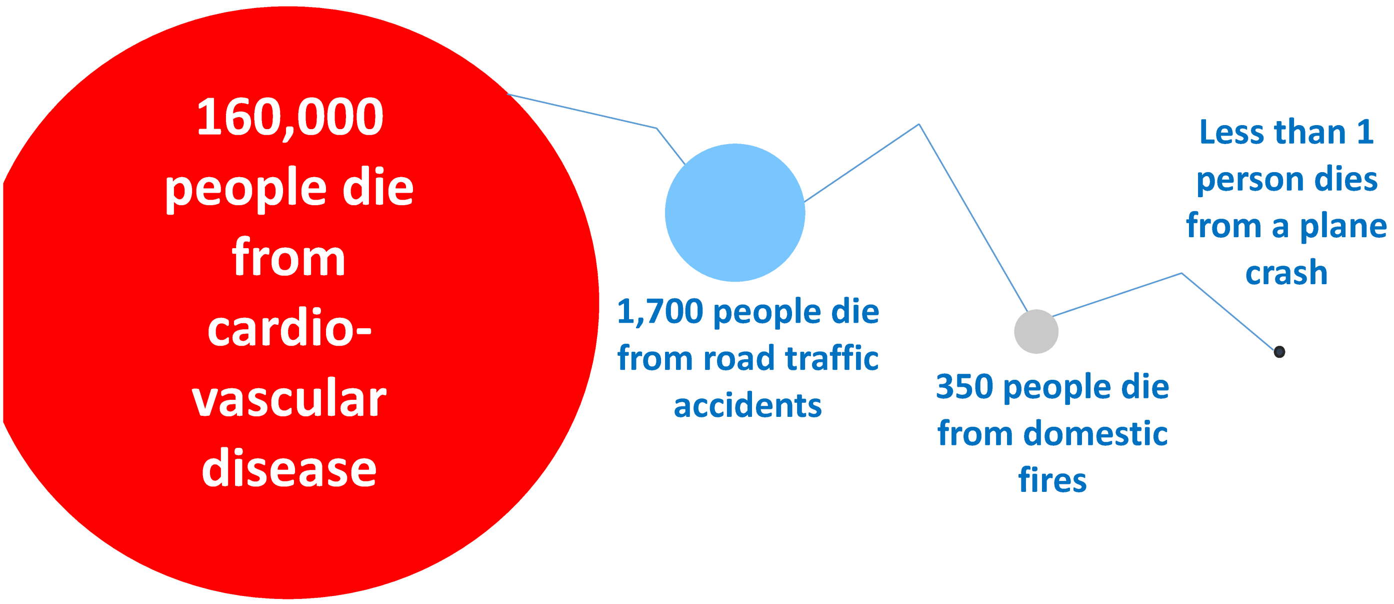

Health Check Tests

Your risk calculations are based on the following results from this health check:

Blood pressure

Your blood pressure measurement was [XXX] / [XX] mmHg (systolic/diastolic)

This means it is:

- ❑ In the healthy range
- ❑ A little high
- ❑ Very high

Cholesterol Level

Your cholesterol ratio was [X] (total cholesterol/HDL)

This means you are considered to be:

- ❑ In the healthy range (*5 or below*)
- ❑ Moderate risk (*5-6*)
- ❑ Higher risk (*above 6*)

If you are higher risk you may need to consider medication to lower your cholesterol.

Diabetes Check

Your glycated haemoglobin (HbA1c) result was [X.X] %

This means that you:

- ❑ Do not have diabetes (*less than 42*)
- ❑ Are at risk of developing diabetes (*42-47*)
- ❑ May have diabetes and will need to have further testing (*48 or over*)

Body Mass Index

Your BMI was [XX]

This means that you are:

White, Black African/Caribbean or other ethnic groups:

- ❑ Healthy weight (*18.5 to less than 25*)
- ❑ Overweight (*25 to less than 30*)
- ❑ Obesity 1 (*30 to less than 35*)
- ❑ Obesity 2 (*35 to less than 40*)
- ❑ Obesity 3 (*40 or more*)

South Asian or other Asian:

- ❑ Healthy weight (*18.5 to less than 23*)
- ❑ Overweight (*23 to less than 27.5*)
- ❑ Obesity 1 (*27.5 to less than 32.5*)
- ❑ Obesity 2 (*32.5 to less than 37.5*)
- ❑ Obesity 3 (*37.5 or more*)

You said you smoke [XX] cigarettes a day

66% of smokers would like to give up the habit. Giving up 20 premium brand cigarettes a day would save approximately £3,000 a year. You are up to four times more likely to quit if you use NHS support than if you go it alone.

All areas have a free local NHS Stop Smoking Service which can help you find your best way of stopping, providing the medication and support you need. Call 0300 123 1044 for more information.

You said you drink [XX] units of alcohol per week

Cutting out a couple of large glasses of wine or beer each week could save you more than £400 year, as well as helping you to lose weight and get better quality sleep.

Drinking within recommended guidelines (no more than 14 units per week for men and women), not regularly drinking more 2-3 units a day, and having at least two alcohol free days per week will reduce your risk.

NHS

Your Personal Action Plan

Now that you know your cardiovascular risk and heart age, what can you do to improve it?

- ☐ Lose some weight

  Sign up to a weight loss programme, cut out foods and drinks with high levels of sugar and fats

- ☐ Eat more healthily

  Eat your 5 a day, swap out sugary drinks for diet or sugar-free versions, choose wholegrain foods such as wholemeal bread and pasta and brown rice, cook using fresh ingredients at home

- ☐ Do more exercise

  Sign up to a sports group or gym, take the stairs instead of lifts, walk and cycle to work

- ☐ Reduce your blood pressure and cholesterol

  Grill, bake, steam and poach meat instead of frying or roasting, eat more fibre (wholegrains such as wholemeal bread and pasta and vegetables), eat less fatty meats, dairy foods, ghee and cakes and biscuits

- ☐ Reduce your salt intake

  Add less salt to your cooking or plate, check labels on ready-made foods

- ☐ Stop smoking

  Order your Smokefree quit kit today, save your cigarette money in a pot to see how it builds up

- ☐ Reduce the amount of alcohol you drink

  Use smaller glasses or measures when pouring wine and spirits at home, have a few drink-free days each week, alternate alcoholic drinks with glasses of water, drink with a meal

What goal(s) would you like to set yourself?

What small changes would help you achieve your goal(s)?

When will you achieve this?

Who can help you achieve this goal?

NHS

For more information and support about how to achieve these goals, visit or call:

- NHS Health Check w: www.nhs.uk/Conditions/nhs-health-check
- NHS free weight loss plan w: www.nhs.uk/Livewell/weight-loss-guide
- Change for life w: www.nhs.uk/change4life t: 0300 123 4567
- Smokefree NHS w: www.nhs.uk/smokefree t: 0300 123 1044
- NHS Alcohol information w: www.nhs.uk/Livewell/alcohol
- Action on Salt w: www.actiononsalt.org.uk t: 020 7882 5941
- Diabetes w: www.diabetes.co.uk
- Dementia w: www.alzheimers.org.uk t: 0300 222 1122

What's good for the heart is good for the head

Treatment:

After this health check, you may need a follow up appointment, and your doctor may prescribe you some medication to manage your risk of cardiovascular disease. You will be told by the clinic staff if you need to make another appointment.

For every 20 potential strokes or heart attacks, treatment will prevent 7:

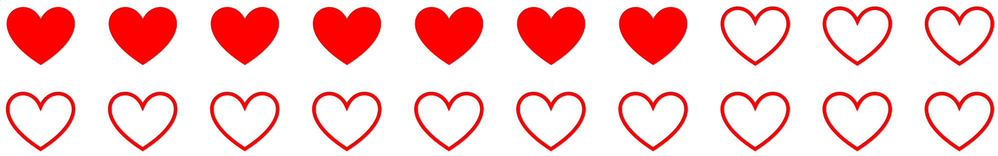

- ☐ Remember to take any prescribed treatment according to your doctor's instructions

  Set the alarm on your phone to remind you or put the pills next to your toothbrush

Extra information/ Local Support Groups:

Your next appointment will be due in…………………………………………………………………………………….

This NHS Health Check Risk Report was devised by the Clinical Effectiveness Group QMUL in collaboration with the East London CVD Prevention team at Bart's Heart Centre. It is licensed under the Creative Commons Attribution-NonCommercial 4.0 International License. To view a copy of this license, visit http://creativecommons.org/licenses/by-nc/4.0/ or send a letter to Creative Commons, PO Box 1866, Mountain View, CA 94042, USA.
