# Supplementary material for: Improving cardiovascular disease risk communication in NHS Health Checks: a qualitative study
Source: BMJ Open. 2019 Sep 3;9(8):e026058. doi: 10.1136/bmjopen-2018-026058 (PMC6731822; doi:10.1136/bmjopen-2018-026058)
Supplement: Supplementary data [file bmjopen-2018-026058supp002.pdf]

Consolidated criteria for reporting qualitative studies (COREQ): 32-item checklist

No	Item	Guide questions/description		Page
**Domain 1: Research team and reflexivity**				
Personal Characteristics				
1.	Interviewer/facilitator	Which author/s conducted the interview or focus group?	MKDH	5
2.	Credentials	What were the researcher's credentials? *E.g. PhD, MD*	MPH, BSc	1
3.	Occupation	What was their occupation at the time of the study?	Research assistant	1
4.	Gender	Was the researcher male or female?	F	5
5.	Experience and training	What experience or training did the researcher have?	Training in qualitative research and previous experience in health related communication	5
Relationship with participants				
6.	Relationship	Was a relationship	No	5

No	Item	Guide questions/description		Page
	established	established prior to study commencement?		
7.	Participant knowledge of the interviewer	What did the participants know about the researcher? e.*g. personal goals, reasons for doing the research*	Participants were informed that MKDH was a university researcher conducting a study	5
8.	Interviewer characteristics	What characteristics were reported about the interviewer/facilitator? e.g. *Bias, assumptions, reasons and interests in the research topic*	Informed MKDH was interested in improving NHS Health Checks	5
**Domain 2: study design**				
Theoretical framework				
9.	Methodological orientation and Theory	What methodological orientation was stated to underpin the study? *e.g. grounded theory, discourse analysis, ethnography, phenomenology, content analysis*	Inductive thematic analysis with coding of transcripts according to latent and manifest content and developing a thematic framework.	6
Participant selection				

No	Item	Guide questions/description		Page
10.	Sampling	How were participants selected? *e.g. purposive, convenience, consecutive, snowball*	Purposive maximum diversity sample	5
11.	Method of approach	How were participants approached? e.*g. face-to-face, telephone, mail, email*	Mail and face to face	5
12.	Sample size	How many participants were in the study?	18	6
13.	Non-participation	How many people refused to participate or dropped out? Reasons?	None	6
Setting				
14.	Setting of data collection	Where was the data collected? e.*g. home, clinic, workplace*	General practice clinic	5
15.	Presence of non-participants	Was anyone else present besides the participants and researchers?	No one	5
16.	Description of sample	What are the important characteristics of the sample? *e.g. demographic data, date*	Demographic	7
Data collection				
17.	Interview guide	Were questions, prompts, guides provided by the authors? Was it pilot tested?	Pilot tested The topic guide [box 1] was piloted, used open questions	5/6

No	Item	Guide questions/description		Page
			and probes to gather more detail, and was adapted as new themes emerged.	
18.	Repeat interviews	Were repeat interviews carried out? If yes, how many?	No	5
19.	Audio/visual recording	Did the research use audio or visual recording to collect the data?	Yes audio recording	5
20.	Field notes	Were field notes made during and/or after the interview or focus group?	Yes	5
21.	Duration	What was the duration of the interviews or focus group?	10-40 minutes	5
22.	Data saturation	Was data saturation discussed?	Yes	5
23.	Transcripts returned	Were transcripts returned to participants for comment and/or correction?	No	5
**Domain 3: analysis and findings**z				
Data analysis				
24.	Number of data	How many data coders	2	6

No	Item	Guide questions/description		Page
	coders	coded the data?		
25.	Description of the coding tree	Did authors provide a description of the coding tree?	No	6
26.	Derivation of themes	Were themes identified in advance or derived from the data?	Derived from data	6
27.	Software	What software, if applicable, was used to manage the data?	NVIVO 10	6
28.	Participant checking	Did participants provide feedback on the findings?	No	6
Reporting				
29.	Quotations presented	Were participant quotations presented to illustrate the themes / findings? Was each quotation identified? e.*g. participant number*	Yes; each participant is identified	7-11
30.	Data and findings consistent	Was there consistency between the data presented and the findings?	Yes	7-11
31.	Clarity of major themes	Were major themes clearly presented in the findings?	Yes	7
32.	Clarity of minor themes	Is there a description of diverse cases or discussion of minor themes?	Yes	11
