# Supplementary material for: Improving cardiovascular disease risk communication in NHS Health Checks: a qualitative study
Source: BMJ Open. 2019 Sep 3;9(8):e026058. doi: 10.1136/bmjopen-2018-026058 (PMC6731822; doi:10.1136/bmjopen-2018-026058)
Supplement: Author's manuscript [file bmjopen-2018-026058.draft_revisions.pdf]

BMJ Open is committed to open peer review. As part of this commitment we make the peer review history of every article we publish publicly available.

When an article is published we post the peer reviewers' comments and the authors' responses online. We also post the versions of the paper that were used during peer review. These are the versions that the peer review comments apply to.

The versions of the paper that follow are the versions that were submitted during the peer review process. They are not the versions of record or the final published versions. They should not be cited or distributed as the published version of this manuscript.

BMJ Open is an open access journal and the full, final, typeset and author-corrected version of record of the manuscript is available on our site with no access controls, subscription charges or pay-per-view fees (http://bmjopen.bmj.com).

If you have any questions on BMJ Open's open peer review process please email info.bmjopen@bmj.com

BMJ Open

Improving cardiovascular disease risk communication in NHS Heath Checks: a qualitative study

Journal:	*BMJ Open*
Manuscript ID	bmjopen-2018-026058
Article Type:	Research
Date Submitted by the Author:	14-Aug-2018
Complete List of Authors:	Hawking, Meredith; Barts and The London School of Medicine and Dentistry, Centre for Primary Care and Public Health<br>Timmis, Adam; Bart's Heart Centre, NIHR Cardiovascular Biomedical Research Unit<br>Wilkins, Fae ; Barts and The London School of Medicine and Dentistry, Queen Mary University of London., Centre for Primary Care and Public Health<br>Potter, Jessica; Barts and The London School of Medicine and Dentistry, Queen Mary University of London, Centre for Primary Care and Public Health<br>Robson, John; Barts and The London School of Medicine and Dentistry, Queen Mary University of London, Centre for Primary Care and Public Health
Keywords:	PUBLIC HEALTH, PREVENTIVE MEDICINE, PRIMARY CARE, Information technology < BIOTECHNOLOGY & BIOINFORMATICS, Communication

SCHOLARONE™
Manuscripts

Improving cardiovascular disease risk communication in NHS Heath Checks: a qualitative study

Authors: Hawking M.K.D, Timmis A, Wilkins F, Potter J.L, Robson J.

Author details

Ms Meredith Kate Durolf Hawking. MPH BSC Hons. Research Fellow[1]
Corresponding author: email: m.k.d.hawking@qmul.ac.uk

Prof Adam Timmis. MA MD FRCP. Professor of Clinical Cardiology[2]
Barts and the London School of Medicine and Dentistry, Barts Heart Centre EC1A 7BE
a.d.timmis@qmul.ac.uk

Ms Fae Wilkins. Facilitator[1]
Centre for Primary Care and Public Health, Queen Mary University London, 58 Turner Street, London E1 2AB
f.wilkins@qmul.ac.uk

Dr Jessica L Potter MBBS BA MRCP. MRC Doctoral Fellow[1]
Centre for Primary Care and Public Health, Queen Mary University London, 58 Turner Street, London E1 2AB
Jessica.potter@qmul.ac.uk

Dr John Robson MD FRCGP. Reader[1]
Centre for Primary Care and Public Health, Queen Mary University London, 58 Turner Street, London E1 2AB
j.robson@qmul.ac.uk

Tel: 0207 882 2553

1) Barts and The London School of Medicine and Dentistry, Queen Mary University London, 58 Turner Street, London E1 2AB

2) Barts and the London School of Medicine and Dentistry, Barts Heart Centre, London, EC1A 7BE

For peer review only

Abstract

Objective

To explore patient perspectives and experiences of a personalised Risk Report, designed to improve cardiovascular risk communication in the NHS Health Check.

Design and Setting

Qualitative study with NHS Health Check attendees in three general practices in the London Borough of Newham.

Intervention and participants

A personalised Risk Report for the NHS Health Check was developed to improve communication of results and advice. The Risk Report was embedded in the electronic health record, printed with auto-filled results, and used as a discussion aide during the NHS Health Check, and was a take-home record of information and advice on risk reduction for the attendees.

18 purposively sampled socially diverse participants took part in semi-structured interviews which were analysed thematically.

Results

For most participants, the NHS Health Check was an opportunity for reassurance and assessment, and the Risk Report was an enduring record that supported risk understanding, with impact beyond the individual. For a minority, ambivalence towards the Risk Report occurred in the context of attending for other reasons, and risk and lifestyle advice were not internalised or acted upon.

Conclusion

Our findings demonstrate the potential of a personalised Risk Report as a useful intervention in NHS Health Checks for enhancing patient understanding of cardiovascular risk and strategies for risk reduction. Also highlighted are the challenges that must be overcome to ensure transferability of these benefits to diverse patient groups.

Strengths and weaknesses

- This study was carried out during routine delivery of the NHS Health Check programme and included socially diverse participants at low or moderate cardiovascular risk.
- The study was limited to delivery in a general practice setting and may not be generalisable to delivery in other settings.
- The risk report was only available in an English language version and may be less suitable for people who prefer another language.

For peer review only

Introduction

The national NHS Health Checks programme, initiated in 2009 across England, is a publicly funded screening and prevention programme aiming to detect early signs of cardiovascular ill health amongst healthy 40-74 year olds in the general population.(1) Effective communication of cardiovascular disease (CVD) risk is a core element of this programme but previous qualitative research has suggested that patients struggle to understand risk in NHS Health Checks(2-4) and are dissatisfied with the lack of information provided.(5) A survey of patients found that over 70% recalled receiving lifestyle advice, but very few remembered receiving a CVD risk score and many incorrectly believed themselves to be low risk.(5) Research on attitudes of attendees and non-attendees called for consistent provision of tailored lifestyle information (6) and cited limited communication of risk and inadequate access to support services as prime concerns relevant across differing age groups, ethnic groups and social groups.(7, 8)

In East London the majority of NHS Health Checks are delivered by trained Health Care Assistants (HCAs), typically multi-lingual staff drawn from local communities.(9) At present in East London there is no formal, standardised mechanism for conveying CVD risk information other than verbal communication during the NHS Health Check itself.

To improve communication in NHS Health Checks, we developed an evidence based personalised NHS Health Check Risk Report, to be used both as a discussion aid and as an enduring record for patients to take away. This incorporated an infographic explanation of their CVD risk as well as findings from clinical tests and a personalised action plan. This paper reports on qualitative findings from a mixed methods feasibility trial of the Risk Report in general practice.

Methods

Development of the Risk Report

The Risk Report (Fig 1) was developed in line with national NHS Health Check programme guidelines and informed by the EAST framework,(10) risk communication literature and focus groups with HCAs, nurses and clinicians who are involved in the provision of Health Checks.

Input on implementation, content and design was gathered from general practitioners, specialist cardiologists and two informal focus groups with HCAs and nurses. Implementation barriers included: limited access to colour printers, high printing costs, and time taken to hand write documents, therefore most patients leave with no record of their results, the goals they have been set, or resources for achieving them. In response, the Risk Report was designed to be embedded and saved within the electronic health record (EHR), to be automatically populated with patient specific clinical information, easily printed off and discussed with the patient, and suitable for patients to take home.

Public participation

The design of the risk report was informed by the literature on client responses to information and risk communication and modified in the light of responses by attendees during development and piloting. We did not directly involve members of the public in the design of the qualitative study or

For peer review only

its analysis. The qualitative interviews were designed to gain insight from participants into the delivery of improved methods of risk communication.

Content of the risk report

We shaped elements of the Risk Report around the four principles of the EAST framework: easy, attractive, social and timely.(10) *Easy*: embedding the Risk Report in the EHR and simple printing, cut 'hassle and time factors'. Complex behavioural goals were simplified by breaking down complex goals into achievable steps and including supporting resources. *Attractive*: NHS branding was used to improve trustworthiness (11, 12) alongside infographics, personalisation and plain English wording. Health benefits of behaviour change were presented with alternative incentives to boost motivation to change. *Social*: Social norms messaging was used to motivate behaviour change and increase salience. The action planning section includes family and friends in goal setting, prompts discussion and encourages making a commitment to others. *Timely*: delivery of messages is optimal when in the appointment setting, but continues as patients reflect on and refer back to the report. Messaging highlights immediate as well as long term benefits of making behavioural changes.

The Risk Report includes QRISK2(13) and Heart Age (62) metrics to convey CVD risk, using infographics, icon arrays and pictorial natural frequencies with a common denominator to ease understanding and reduce denominator neglect.(14-17) Risk messages include a temporal component to provide context.(18) An infographic comparing comparative 'dread' risks is included to help patients situate their CVD risk alongside other causes of mortality.(19) We used survival framing to encourage risk-averse choices in terms of medication taking (20) and positive framing of messages to highlight alternative benefits of making healthy behaviours.(21) The Risk Report is printed in greyscale on four sides of A4 and includes information on local lifestyle change support services. It is available on-line at https://www.qmul.ac.uk/blizard/ceg/resource-library/presentations/nhs-health-check-risk-report-oct-2017/

[Insert fig 1 image of part of risk report]

Study design

Nested qualitative interview study within a randomised feasibility trial. The study methods for the trial have been reported separately *(linked trial report)*.

Recruitment and selection

Participants were recruited from three practices in the London Borough of Newham. Study information, consent form and an invitation to attend the NHS Health Check were provided in advance of recruitment. Participants gave written informed consent to take part in a qualitative interview at enrolment into the feasibility trial, and verbally prior to interview commencement.

Participants in the trial who had received the Risk Report within an NHS Health Check were purposively sampled following a maximum diversity sampling approach, according to four categories: age, gender, ethnic group and CVD risk score, and were then invited to interview. They were telephoned by the project administrator or a practice receptionist to schedule their appointment.

For peer review only

Data collection

The development of the topic guide [Box 1] was based on a literature review and was piloted at one of the practices. The topic guide used open questions and probes to gather more detail, and was adapted as new themes emerged. Single instance semi-structured interviews were undertaken in English, face to face in a private room at the practice by an experienced qualitative researcher (MKDH) with a background in public health and health communication. Participants no contact with the researcher before interview, and MKDH was introduced as a university researcher interested in improving NHS Health Checks. The Risk Report was introduced part way through the interview as a prompt and prop for discussion. Sessions lasted 10-40 minutes and were audio recorded, pseudonymised and then transcribed. Field notes were taken after each interview. Data collection occurred March 2016 – July 2017. Interviews commenced until saturation occurred and no new themes were arising.(22) Interviewees received a £20 shopping voucher for their time.

Box 1: Topic Guide

- Early impressions of the NHS Health Check programme
- Motivations to attend/ accessibility
- Overall experience
- Key information/ messages
- Nurse/HCA communication
- Understanding risk

---Introduce Risk Report---

- General feedback (first impressions/ comprehensiveness etc.)
- Design
- Suggestions for improvement
- Risk information
- Lifestyle changes
- Further comments

Analysis

Inductive thematic analysis of the qualitative data was undertaken,(23) which involved coding the transcripts according to latent and manifest content and developing a thematic framework.(23) Analysis by MKDH proceeded alongside data collection and NVIVO 10 software was used for data management.(24) 10% of transcripts were double coded by a second experienced qualitative researcher (JP). Attention was paid to the development of narratives within and across transcripts. Any inconsistencies were discussed and agreed amongst the research team to offer multiple perspectives on the development of codes and identification of themes, and overarching thematic connections.

Results

Sample Characteristics

18 respondents were well represented across gender, age group and self-defined ethnic group [Table 1]. Respondents at lower cardiovascular risk (QRisk2 score) were well represented but those at high risk were not represented in the sample.

Group	Gender		Age Group (years)			QRISK2 score (%)			Ethnicity by grouping			
	Male	Female	40-50	51-60	60-74	10 or less	10 to 19	20 or more	White (British, European)	Black (British/ African or Caribbean)	South Asian (Indian, Pakistani, Bangladeshi, Sri Lankan)	All other Ethnicities
Control	3	4	2	4	1	6	1	0	0	3	2	2
Intervention	9	3	2	5	4	5	6	0	2	5	4	0
**Totals**	**11**	**7**	**4**	**9**	**5**	**11**	**7**	**0**	**2**	**8**	**6**	**2**

Table 1. Characteristics of sample

Overview of themes

Our thematic framework resulted in the identification of four main themes, which are outlined in Table 2. On further interpretation we recognised two overarching threads in our data. These were patterns in the linking of themes. We discuss the findings within each theme and sub-theme before discussing how the themes link together.

Table 2 – Thematic framework

Differing Motivations	Multiple risks	Risk Report as an enduring record	Impact
	Binary risk<br>Risk meanings		
Detecting disease	Acceptance of risk	'Proof' of results	Making changes
Being a willing patient	Rejection of risk	Ambivalency	Not making changes

1. Differing Motivations

Detecting disease

Most participant accounts of why they attended the NHS Health Check were underscored by a desire to check their fitness, or to screen for previously undetected disease, especially in the context of increasing age. This was framed as ‘maintaining your health’

> *“C101: Because I, I’m over 50 now, so I guess now is the right time to say you will have a check on your health, and try and see how fit you are.”*

There was a sense that some interviewees wanted to be reassured ‘just in case’, with reported fears linked to having a family history of CVD or previous test results, such as high blood pressure results.

Being a willing patient

Amongst a minority of interviewees, the reason they attended was out of a sense of obligation to the practice and to the NHS more generally. One participant wanted to be seen as a ‘willing patient’. Another attended to make sure they did not get removed from the practice register.

> *“C103: Probably to keep in with the practice, you know, showing that I was a good, energetic, willing patient.”*

2. Multiple risks

There were multiple constructions of ‘risk’ in the interviews, reflecting the differences in participants’ perceptions of their own health, whether they were concerned and whether or not there were language barriers during the health check.

Binary risk

Those for whom English was not a first language mostly communicated their CVD risk in general, binary terms when asked, reporting that they were ‘fine’ or ‘not at risk’. Risk for these interviewees was either a state of being ‘at risk’, or not. Percentage risk and/or heart age were not mentioned or discussed.

Risk meanings

Those that discussed risk in terms of their risk scores applied different meanings and importance to QRisk and heart age. For instance, this participant was explaining how she would like to have known her QRisk score, because the two types of risk have different meanings for her:

> *“C202: This [heart age] is good to look at, it makes me feel young and things like, oh yeah I’m healthy-ish but our heart age doesn’t, it’s nice but I would have liked to have known my [Q] risk, e… I think it would make you make more changes when you can actually see, OK, so I’m really at risk here […] because even though it’s 44 yeah, I don’t know the risks.”*

Most participants were quicker to remember and report their heart age spontaneously in comparison to their QRISK2 score.

For peer review only

Acceptance of risk

Interviewees reacted to their CVD risk scores in varied ways based on their perceptions of their state of health before attending the check. Those who mentioned being concerned about their CVD health prior to attending the NHS Health Check were more likely to report their risk score and accept it as a 'true' reflection of their current state of health:

> *"C204: And she told me about the age, like my heart is 54 years old when I'm just 51. Yeah, you don't reach, I wanted it to be lower […] I'm going to try my best to bring it lower. That's what I'm trying to do right now."*

Rejection of risk

Some participants already believed themselves to lead a healthy life or have a healthy heart and this meant that they disagreed with or discounted the CVD risk that was presented to them in the check if it did not fall in line with their own perception. For them, their risk was pre-defined by how they felt and saw themselves prior to the check:

> *"C103: Yeah, now that was a bit odd actually because the first time she said that there was a 1 in 10 chance of me having a heart attack and I thought, well that just doesn't seem right, once I got home I thought, no, that really doesn't seem right […] So that was a bit odd but I thought, I'm not going to have one [a CVD event] anyway. No. [laughs]"*

For these participants, their perception of not being 'at risk' was based on feeling healthy and leading a healthy life, a lack of symptoms or the belief that good health is bestowed by a higher power.

3. Risk Report as an enduring record

The Risk Report prompted participants to recall their CVD risk results. They found it to be 'user friendly' and clear with a good size font, no jargon and good for people for whom English was not their first language.

'Proof' of results

The majority of participants talked positively about the Risk Report as an enduring record of their results:

> *"C202: I think I liked the bit of paper that they gave me at the end that just jots down everything because I think you forget really easily and that's been good to look back at."*

For some, this had additional meaning, as 'proof' and reassurance of their good health, as this quote describes:

> *"C109: Having something positive as proof on a piece of paper, like you could actually, a physical piece of paper. I know it sounds silly right but having a, it's like, hey if the pain is still there, like I say, at least my heart's working properly."*

For peer review only

Participants reported looking back at the Risk Report to jog their memory. Some shared and discussed it with family and friends. Two reported keeping it by their bed, and some discussed how they would keep it to compare their results in future checks.

> *"C207: Some leaflets you can look at and throw it away but when you look at that box and you're look at it and you're thinking, oh yeah that's interesting. […] Yeah, it does motivate you, as it has for me anyway, it really has."*

Ambivalency

A few interviewees were not interested in the Risk Report as a record at all, had not looked back at it or engaged with it:

> *"I: Did you get a leaflet [showing Risk Report] like that?*
>
> *C205: Might be. I haven't opened it since. It might still be in the house."*

4. Impact

Making changes

Most patients reported making at least small changes as a result of the NHS Health Check, such as incorporating more walks, reducing their salt and fat intake, and cutting down on alcohol consumption. A few participants had made significant changes – for instance one patient had begun exercising regularly, joined a gym with his partner, cut down on fried foods, avoided drinking strong spirits and begun smoking less, reporting his cholesterol reduced from 5.5 to 4.9 in three months.

Not making changes

There were a few patients who had not made any changes to their lifestyle after the Check. These tended to be patients who reported that they were low risk or who already felt they were in good health. One patient, who had a medium QRisk2 score, was pre-diabetic and had been referred on for further tests, had tried to make changes but did not manage to keep it up:

> *"C104: Well I want to but you've not got the go all the time, sometimes you just want to binge, what I do a lot. […] The diet plans, my son printed some sheets off from work, what you're supposed to be eating, but you'd do it for a few days and then you put them in a drawer."*

Another patient had also been referred on for more tests to investigate his glucose levels but had not attended the referral clinic. He clearly understood his risk and what had been asked of him, however did not change his behavior according to the NHS Health Check results and recommendations:

> *"C205: I was supposed to go for that check up […] And I didn't go for that. […] As long as I can still run and walk and I don't have pains and aches and dizzy spells as they like to call them. I don't. I don't worry."*

Wider impact

For peer review only

It was clear from the interviews that there was a wider social impact of NHS Health Checks that went beyond individuals. This included sharing the information from the Check with others, family members being included in diet and exercise changes, as well as recommendations to family and friends that they should attend their NHS Health Check.

> *"C202: So now I have to look at my diet and actually even for my children as well, and look at their diet and just think, is it necessary, do we need all this salt, and I do really like salt and we are like a salty family. I would just add salt to everything and now I'm starting to think that I can't."*

> *"C207: I showed her [wife] and yeah and I've looked at it and it's when we go shopping now we, we probably have more vegetables and fruits in our shopping trolley than we have of all crisps."*

5. Bringing the themes together

The majority of participants' accounts played out according to the main thematic findings described above. Participants attended their NHS Health Check to assess their levels of fitness and to get reassurance that nothing was wrong, or because they were concerned about previous tests or predisposition to ill-health. They accepted the level of risk that the screening assigned to them, and viewed the Risk Report as a positive enduring record of their health status. They then went on to make lifestyle changes and share these with their families.

A minority of participant accounts in the study followed a different narrative from the central themes. These participants attended out of obligation or duty rather than concern for their health. They did not readily accept or internalise risk scores that differed from their own assessments of being healthy. They were ambivalent towards the Risk Report and did not make advised lifestyle changes. These diverse cases were few but are important for our consideration and recognition.

Discussion

Summary

For most, the NHS Health Check was an opportunity for reassurance and assessment, and the Risk Report was an enduring record that supported risk understanding, and supported lifestyle change for the individual and their wider social networks. For a minority, ambivalence towards the Risk Report occurred in the context of attending for other reasons, and risk and lifestyle advice were not internalised or acted upon.

Strengths and Limitations

Our sample included medium and low CVD risk participants from a range of ethnic groups and ages, reflecting the Newham population, of whom the majority are low to medium risk.(9) Many low to medium risk patients are unlikely to receive further clinical referrals as a result of the NHS Health Check, and as such, the Risk Report may be the only intervention that they receive.

For peer review only

Our findings may not be transferrable to those who do not take up the offer of the NHS Health Check, nor those who do not speak English. The inclusion of four patients for whom English was not their first language was pragmatic and reflects routine care in many areas, where health care professionals must communicate with patients about CVD risk regardless of language barriers and without the assistance of professional translators. Specific adaptations to the Risk Report and the NHS Health Check programme are required to extend the programme more equitably to all types of patient groups.

Comparison with Existing literature

Familial experience with CVD (25) and concerns about preventable or undetected illness as motivators to attend (26, 27) have been reported elsewhere. Some participants in our study presented due to specific concerns, such as high blood pressure, supporting Perry's assertion that it is not only the 'worried well' who attend.(4) Burgess and colleagues (28) found civic responsibility to be a driver for some patients, which falls in line with being seen as a 'willing patient'. This study builds on these findings by aligning motivations to attend with the ongoing experiences of having a health check.

One key finding was the notion of CVD risk as a binary category, rather than a percentage scale. The 'take home message' from the NHS Health Check was often limited to dualistic generalities like being 'fine' or 'not at risk'. Whether people at higher CVD risk receive meaningful information and people at lower CVD risk are not falsely reassured should be a key focus of research in this area. The concept of an "MOT", or 'roadworthiness'(29) - used prominently in the advertising of some NHS Checks - in which some people 'pass' – and presumably need take no further action - and the others 'fail' may not be the most effective message to convey. van Steenkiste and colleagues (30) found that communicated test results faded into an 'overall reassuring message' instead of specific results. Numerically, most heart attacks and strokes occur in people at moderate CVD risk, so if health checks are to be successful the focus must remain on the importance of reducing the burden of CVD in all patients, not just those at high risk. Challenging the 'MOT' metaphor associated with the overall programme may also help in this regard.

A key finding of our study was the ways in which patients used the Risk Report to not only motivate their own lifestyle changes, but as a way to prompt and support discussions and changes with others in their family and broader social networks. This finding has been mirrored in ethnographic work in a community health check setting, where Afro-Caribbean participants went on to discuss their results with peers.(25) In our study the 'work' of becoming healthier was a shared endeavor, involving those around the 'patient', including older relatives and children. Information impacted beyond the person through social practices such as eating and being physically active. Moving away from an individualistic approach to examine shared practices in the aftermath of health checks may be a fruitful area for future research.

The majority of participant views aligned with the dominant biomedical risk narrative behind screening for preventable illness in the UK. This viewpoint places the responsibility for maintaining health to the individual, via 'correct' lifestyle choices.(31) Having an NHS Health Check feeds this narrative by offering the opportunity for reassurance and provision of guidance to make small changes to maintain or protect health from uncertainty, or risk. However, we found a minority of participants did not engage with the risk element of the NHS Health Checks, or intend to make

For peer review only

lifestyle changes as a result of having a check, instead they attended for other reasons and were ambivalent about the risk report and the idea of 'improving' their health. These patients are often characterised as 'non-attenders' without an attempt to explore their worldview.(32, 33) Models of continuous care, characterised by trusting clinician-patient relationships (34) and attention to patient narratives (35) may allow for better ways of communicating about cardiovascular ill-health with patients whose perspectives do not align with the dominant biomedical narrative. Additionally, qualitative attention to the whole patient and their journey through the NHS Health Check and beyond could help identify alternative ways of delivering good care for these groups of patients.

Implications for research and practice

Our qualitative results support further development of a Risk Report for NHS Health Checks, to enhance risk communication and support lifestyle modification and dissemination of messages amongst wider social networks. Our findings also highlight some of the challenges faced by the NHS Health Check programme for supporting patients whose ideas about risk and screening for future illness don't align with NHS and preventative public health priorities. Shifting the focus of the NHS Health Checks beyond the individual, to consider how networks of family and friends might interact, may have positive influences for leading healthier lives.

Additional information

Funding

This study was funded by The Guttmann Academic Partnership hosted by UCLPartners. Meredith K.D. Hawking was in part supported by the National Institute for Health Research (NIHR) Collaboration for Leadership in Applied Health Research and Care (CLAHRC) North Thames at Bart's Health NHS Trust.

Ethical Approval

The study received final approval from the National Research Ethics Service Committee North West (Preston) on 7/8/2015. The research ethics committee reference was: 15/NW/0635 (protocol number MCPH1C8R). The study was registered with ClinicalTrials.gov (NCT02486913).

Competing Interests

MKDH, JLP, AT and FW have no interests to declare. JR has a non-pecuniary interest as a co-author of QRisk2 and also a member of a national NHS Health Check advisory group.

Acknowledgements

We are grateful to the NHS Health Check attendees for giving up their time and sharing their experiences and for the cooperation and support from general practitioners and the clinic staff who were involved in the study and those who helped inform the development of the Risk Report.

Contributorship

For peer review only

MKHD led the design of the risk report and with AT and JR designed the qualitative study. FW organised the recruitment of practices, staff training and participant attendance. MHKD carried out the interviews and analysed the transcripts with JLP. All authors contributed to the manuscript.

References

1. DOH. Putting Prevention First – NHS Health Check: Vascular Risk Assessment and Management Best Practice Guidance. London: Department of Health; 2009.

2. Baker C, Loughren EA, Crone D, Kallfa N. Patients' perceptions of a NHS Health Check in the primary care setting. Quality in primary care. 2014;22(5):232-7.

3. Riley R, Coghill N, Montgomery A, Feder G, Horwood J. Experiences of patients and healthcare professionals of NHS cardiovascular health checks: a qualitative study. Journal of public health (Oxford, England). 2016;38(3):543-51.

4. Perry C, Thurston M, Alford S, Cushing J, Panter L. The NHS health check programme in England: a qualitative study. Health promotion international. 2016;31(1):106-15.

5. Krska J, du Plessis R, Chellaswamy H. Views and experiences of the NHS Health Check provided by general medical practices: cross-sectional survey in high-risk patients. Journal of Public Health. 2015;37(2):210-7.

6. McNaughton RJ, Shucksmith J. Reasons for (non)compliance with intervention following identification of 'high-risk' status in the NHS Health Check programme. Journal of public health (Oxford, England). 2015;37(2):218-25.

7. Ismail H, Atkin K. The NHS Health Check programme: insights from a qualitative study of patients. Health Expect. 2016;19(2):345-55.

8. Shaw RL, Lowe H, Holland C, Pattison H, Cooke R. GPs' perspectives on managing the NHS Health Check in primary care: a qualitative evaluation of implementation in one area of England. BMJ open. 2016;6(7):e010951.

9. Robson J, Dostal I, Madurasinghe V, Sheikh A, Hull S, Boomla K, et al. The NHS Health Check programme: implementation in east London 2009–2011. BMJ open. 2015;5(4).

10. Service OH, M.; Halpern, D.; Algate, F.; Gallagher, R.; Nguyen, S.; Ruda, S.; Sanders, M.; Pelenur, M.; Gyani, A.; Harper, H.; Reinhard, J.; Kirkman, E. EAST: Four ways to apply behavioural insights. London: Behavioural Insights Ltd; 2014.

11. Powell J, Inglis N, Ronnie J, Large S. The Characteristics and Motivations of Online Health Information Seekers: Cross-Sectional Survey and Qualitative Interview Study. J Med Internet Res. 2011;13(1):e20.

12. Quiroz-Aitken M, Cunningham-Burley S, Pagliari C. Moving from Trust to Trustworthiness: Experiences of public engagement in the Scottish Health Informatics Programme. Science and Public Policy. 2016.

13. Hippisley-Cox J, Coupland C, Vinogradova Y, Robson J, Minhas R, Sheikh A, et al. Predicting cardiovascular risk in England and Wales: prospective derivation and validation of QRISK2. BMJ. 2008;336(7659):1475.

For peer review only

14. Kurz-Milcke E, Gigerenzer G, Martignon L. Transparency in risk communication: graphical and analog tools. Annals of the New York Academy of Sciences. 2008;1128:18-28.

15. Goodyear-Smith F, Arroll B, Chan L, Jackson R, Wells S, Kenealy T. Patients prefer pictures to numbers to express cardiovascular benefit from treatment. Annals of family medicine. 2008;6(3):213-7.

16. Garcia-Retamero R, Galesic M, Gigerenzer G. Do icon arrays help reduce denominator neglect? Med Decis Making. 2010;30(6):672-84.

17. Galesic M, Garcia-Retamero R, Gigerenzer G. Using icon arrays to communicate medical risks: overcoming low numeracy. Health psychology : official journal of the Division of Health Psychology, American Psychological Association. 2009;28(2):210-6.

18. Waldron CA, van der Weijden T, Ludt S, Gallacher J, Elwyn G. What are effective strategies to communicate cardiovascular risk information to patients? A systematic review. Patient Educ Couns. 2011;82(2):169-81.

19. Gigerenzer G. Dread risk, September 11, and fatal traffic accidents. Psychological science. 2004;15(4):286-7.

20. Veldwijk J, Essers BAB, Lambooij MS, Dirksen CD, Smit HA, de Wit GA. Survival or Mortality: Does Risk Attribute Framing Influence Decision-Making Behavior in a Discrete Choice Experiment? Value in Health. 2016;19(2):202-9.

21. Akl EA, Oxman AD, Herrin J, Vist GE, Terrenato I, Sperati F, et al. Framing of health information messages. The Cochrane database of systematic reviews. 2011(12):Cd006777.

22. Mason M. Sample Size and Saturation in PhD Studies Using Qualitative Interviews. 2010. 2010;11(3).

23. Braun V, Clarke V. Using thematic analysis in psychology. Qualitative Research in Psychology. 2006;3(2):77-101.

24. QSR, International, Pty, Ltd. NVivo Qualitative Data Analysis Software Version 10. 2012.

25. Riley R, Coghill N, Montgomery A, Feder G, Horwood J. The provision of NHS health checks in a community setting: an ethnographic account. BMC health services research. 2015;15:546.

26. Ellis N, Gidlow C, Cowap L, Randall J, Iqbal Z, Kumar J. A qualitative investigation of non-response in NHS health checks. Archives of Public Health. 2015;73(1):1-8.

27. Jenkinson CE, Asprey A, Clark CE, Richards SH. Patients' willingness to attend the NHS cardiovascular health checks in primary care: a qualitative interview study. BMC Fam Pract. 2015;16:33.

28. Burgess C, Wright AJ, Forster AS, Dodhia H, Miller J, Fuller F, et al. Influences on individuals' decisions to take up the offer of a health check: a qualitative study. Health Expect. 2015;18(6):2437-48.

29. Shaw RL, Pattison HM, Holland C, Cooke R. Be SMART: examining the experience of implementing the NHS Health Check in UK primary care. BMC Fam Pract. 2015;16:1.

30. van Steenkiste B, van der Weijden T, Timmermans D, Vaes J, Stoffers J, Grol R. Patients' ideas, fears and expectations of their coronary risk: barriers for primary prevention. Patient Education and Counseling. 2004;55(2):301-7.

31. Lupton D. Risk as Moral Danger: The Social and Political Functions of Risk Discourse in Public Health. International Journal of Health Services. 1993;23(3):425-35.

For peer review only

32. Dalton A, Bottle R, Okoro C, Majeed F, Millett C. Uptake of the NHS health checks programme in a deprived, culturally diverse setting: cross sectional study. J Epidemiol Commun H. 2011;65.

33. Dryden R, Williams B, McCowan C, Themessl-Huber M. What do we know about who does and does not attend general health checks? findings from a narrative scoping review. BMC Public Health. 2012;12.

34. Robb N, Greenhalgh T. “You have to cover up the words of the doctor”: The mediation of trust in interpreted consultations in primary care. Journal of Health Organization and Management. 2006;20(5):434-55.

35. Greenhalgh T, Collard A, Campbell-Richards D, Vijayaraghavan S, Malik F, Morris J, et al. Storylines of Self-Management: Narratives of People with Diabetes from a Multiethnic Inner City Population. Journal of Health Services Research & Policy. 2011;16(1):37-43.

For peer review only

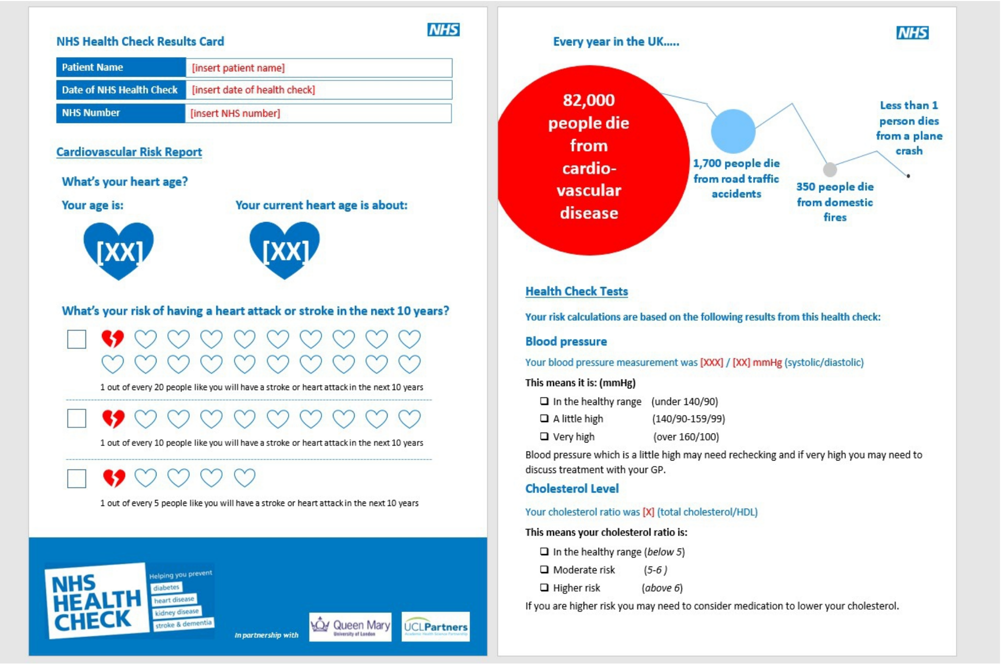

Figure 1. Section of risk report

337x223mm (96 x 96 DPI)

Table 1

Consolidated criteria for reporting qualitative studies (COREQ): 32-item checklist

No	Item	Guide questions/description	
**Domain 1: Research team and reflexivity**			
Personal Characteristics			
1.	Interviewer/facilitator	Which author/s conducted the interview or focus group?	MKHD
2.	Credentials	What were the researcher's credentials? *E.g. PhD, MD*	MPH, BSc
3.	Occupation	What was their occupation at the time of the study?	Research assistant
4.	Gender	Was the researcher male or female?	F
5.	Experience and training	What experience or training did the researcher have?	Training in qualitative research and previous experience in health related communication
Relationship with participants			
6.	Relationship established	Was a relationship established prior to study commencement?	No

For peer review only

No	Item	Guide questions/description	
7.	Participant knowledge of the interviewer	What did the participants know about the researcher? e.g. *personal goals, reasons for doing the research*	Participants were informed that MKHD was a university researcher conducting a study
8.	Interviewer characteristics	What characteristics were reported about the interviewer/facilitator? e.g. *Bias, assumptions, reasons and interests in the research topic*	Informed MKHD was interested in improving NHS Health Checks
**Domain 2: study design**			
Theoretical framework			
9.	Methodological orientation and Theory	What methodological orientation was stated to underpin the study? *e.g. grounded theory, discourse analysis, ethnography, phenomenology, content analysis*	Inductive thematic analysis with coding of transcripts according to latent and manifest content and developing a thematic framework.
Participant selection			
10.	Sampling	How were participants selected? *e.g. purposive, convenience, consecutive, snowball*	Purposive maximum diversity sample

For peer review only

No	Item	Guide questions/description	
11.	Method of approach	How were participants approached? e.g. *face-to-face, telephone, mail, email*	Mail and face to face
12.	Sample size	How many participants were in the study?	18
13.	Non-participation	How many people refused to participate or dropped out? Reasons?	None
Setting			
14.	Setting of data collection	Where was the data collected? e.g. *home, clinic, workplace*	General practice clinic
15.	Presence of non-participants	Was anyone else present besides the participants and researchers?	No one
16.	Description of sample	What are the important characteristics of the sample? e.g. *demographic data, date*	Demographic
Data collection			
17.	Interview guide	Were questions, prompts, guides provided by the authors? Was it pilot tested?	Pilot tested The topic guide used open questions and probes to gather more detail, and was adapted as new themes emerged. Single instance semi-structured

For peer review only

No	Item	Guide questions/description	
			interviews were undertaken in English
18.	Repeat interviews	Were repeat interviews carried out? If yes, how many?	No
19.	Audio/visual recording	Did the research use audio or visual recording to collect the data?	Yes audio recording
20.	Field notes	Were field notes made during and/or after the interview or focus group?	Yes
21.	Duration	What was the duration of the interviews or focus group?	10-40 minutes
22.	Data saturation	Was data saturation discussed?	Yes
23.	Transcripts returned	Were transcripts returned to participants for comment and/or correction?	No
**Domain 3: analysis and findings**z			
Data analysis			
24.	Number of data coders	How many data coders coded the data?	2
25.	Description of the coding tree	Did authors provide a description of the coding tree?	Yes

For peer review only

No	Item	Guide questions/description	
26.	Derivation of themes	Were themes identified in advance or derived from the data?	Yes
27.	Software	What software, if applicable, was used to manage the data?	NVIVO 10
28.	Participant checking	Did participants provide feedback on the findings?	No
Reporting			
29.	Quotations presented	Were participant quotations presented to illustrate the themes / findings? Was each quotation identified? e.g. *participant number*	Yes; each participant is identified
30.	Data and findings consistent	Was there consistency between the data presented and the findings?	Yes
31.	Clarity of major themes	Were major themes clearly presented in the findings?	Yes
32.	Clarity of minor themes	Is there a description of diverse cases or discussion of minor themes?	Yes

For peer review only

BMJ Open

Improving cardiovascular disease risk communication in NHS Heath Checks: a qualitative study

Journal:	*BMJ Open*
Manuscript ID	bmjopen-2018-026058.R1
Article Type:	Research
Date Submitted by the Author:	25-Mar-2019
Complete List of Authors:	Hawking, Meredith; Barts and The London School of Medicine and Dentistry, Centre for Primary Care and Public Health<br>Timmis, Adam; Bart's Heart Centre, NIHR Cardiovascular Biomedical Research Unit<br>Wilkins, Fae ; Barts and The London School of Medicine and Dentistry, Queen Mary University of London., Centre for Primary Care and Public Health<br>Potter, Jessica; Barts and The London School of Medicine and Dentistry, Queen Mary University of London, Centre for Primary Care and Public Health<br>Robson, John; Barts and The London School of Medicine and Dentistry, Queen Mary University of London, Centre for Primary Care and Public Health
**Primary Subject Heading**:	Public health
Secondary Subject Heading:	Communication, Qualitative research, Public health, General practice / Family practice, Cardiovascular medicine
Keywords:	PUBLIC HEALTH, PREVENTIVE MEDICINE, PRIMARY CARE, Information technology < BIOTECHNOLOGY & BIOINFORMATICS, Communication

SCHOLARONE™
Manuscripts

Improving cardiovascular disease risk communication in NHS Heath Checks: a qualitative study

Authors: Hawking M.K.D, Timmis A, Wilkins F, Potter J.L, Robson J.

Author details

Ms Meredith Kate Durolf Hawking. MPH BSC Hons. Research Fellow[1]
Corresponding author: email: m.k.d.hawking@qmul.ac.uk

Prof Adam Timmis. MA MD FRCP. Professor of Clinical Cardiology[2]
Barts and the London School of Medicine and Dentistry, Barts Heart Centre EC1A 7BE
a.d.timmis@qmul.ac.uk

Ms Fae Wilkins. Facilitator[1]
Centre for Primary Care and Public Health, Queen Mary University London, 58 Turner Street, London E1 2AB
f.wilkins@qmul.ac.uk

Dr Jessica L Potter MBBS BA MRCP. MRC Doctoral Fellow[1]
Centre for Primary Care and Public Health, Queen Mary University London, 58 Turner Street, London E1 2AB
Jessica.potter@qmul.ac.uk

Dr John Robson MD FRCGP. Reader[1]
Centre for Primary Care and Public Health, Queen Mary University London, 58 Turner Street, London E1 2AB
j.robson@qmul.ac.uk

Tel: 0207 882 2553

1) Barts and The London School of Medicine and Dentistry, Queen Mary University London, 58 Turner Street, London E1 2AB

2) Barts and the London School of Medicine and Dentistry, Barts Heart Centre, London, EC1A 7BE

For peer review only

For peer review only - http://bmjopen.bmj.com/site/about/guidelines.xhtml

Abstract

Objective

The NHS Health Check programme is a public screening and prevention initiative in England to detect early signs of cardiovascular ill health amongst healthy adults. We aimed to explore patient perspectives and experiences of a personalised Risk Report, designed to improve cardiovascular risk communication in the NHS Health Check.

Design and Setting

Qualitative study with NHS Health Check attendees in three general practices in the London Borough of Newham.

Intervention and participants

A personalised Risk Report for the NHS Health Check was developed to improve communication of results and advice. The Risk Report was embedded in the electronic health record, printed with auto-filled results, and used as a discussion aide during the NHS Health Check, and was a take-home record of information and advice on risk reduction for the attendees.

18 purposively sampled socially diverse participants took part in semi-structured interviews which were analysed thematically.

Results

For most participants, the NHS Health Check was an opportunity for reassurance and assessment, and the Risk Report was an enduring record that supported risk understanding, with impact beyond the individual. For a minority, ambivalence towards the Risk Report occurred in the context of attending for other reasons, and risk and lifestyle advice were not internalised or acted upon.

Conclusion

Our findings demonstrate the potential of a personalised Risk Report as a useful intervention in NHS Health Checks for enhancing patient understanding of cardiovascular risk and strategies for risk reduction. Also highlighted are the challenges that must be overcome to ensure transferability of these benefits to diverse patient groups.

Strengths and weaknesses

- This study was carried out during routine delivery of the NHS Health Check programme and included socially diverse participants at low or moderate cardiovascular risk.
- The study was limited to delivery in a general practice setting and may not be transferable to delivery in other settings.
- The risk report was only available in an English language version and may be less suitable for people who prefer another language.

For peer review only - http://bmjopen.bmj.com/site/about/guidelines.xhtml

Introduction

The national NHS Health Checks programme, initiated in 2009 across England, is a publicly funded screening and prevention programme aiming to detect early signs of cardiovascular ill health amongst healthy 40-74 year olds in the general population.(1) Effective communication of cardiovascular disease (CVD) risk is a core element of this programme but previous qualitative research has suggested that patients struggle to understand risk in NHS Health Checks(2-4) and are dissatisfied with the lack of information provided.(5) A survey of patients found that over 70% recalled receiving lifestyle advice, but very few remembered receiving a CVD risk score and many incorrectly believed themselves to be low risk.(5) Research on attitudes of attendees and non-attendees called for consistent provision of tailored lifestyle information (6) and cited limited communication of risk and inadequate access to support services as prime concerns relevant across differing age groups, ethnic groups and social groups.(7, 8)

In East London the majority of NHS Health Checks are delivered by trained Health Care Assistants (HCAs), typically multi-lingual staff drawn from local communities.(9) At present in East London there is no formal, standardised mechanism for conveying CVD risk information other than verbal communication during the NHS Health Check itself.

To improve communication in NHS Health Checks, we developed an evidence based personalised NHS Health Check Risk Report, to be used both as a discussion aid and as an enduring record for patients to take away. This incorporated an infographic explanation of their CVD risk as well as findings from clinical tests and a personalised action plan. This paper reports on qualitative findings from a mixed methods feasibility trial of the Risk Report in general practice.

Methods

Development of the Risk Report

The Risk Report (figure 1) was developed in line with national NHS Health Check programme guidelines and informed by the EAST framework,(10) risk communication literature and focus groups with HCAs, nurses and clinicians who are involved in the provision of Health Checks.

Input on implementation, content and design was gathered from general practitioners, specialist cardiologists and two informal focus groups with HCAs and nurses. Implementation barriers included: limited access to colour printers, high printing costs, and time taken to hand write documents, therefore most patients leave with no record of their results, the goals they have been set, or resources for achieving them. In response, the Risk Report was designed to be embedded and saved within the electronic health record (EHR), to be automatically populated with patient specific clinical information, easily printed off and discussed with the patient, and suitable for patients to take home.

Public involvement

Patients/ the public were not directly involved in the research planning or delivery process, including research question, recruitment, data collection or analysis. The design of the risk report was

For peer review only - http://bmjopen.bmj.com/site/about/guidelines.xhtml

informed by the literature on client responses to information and risk communication and, as this was a feasibility study, modified in the light of responses by study participants. The qualitative interviews were designed to gain insight from participants into the delivery of improved methods of risk communication.

Content of the Risk Report

We shaped elements of the Risk Report around the four principles of the EAST framework: easy, attractive, social and timely.(10) *Easy*: embedding the Risk Report in the EHR and simple printing, cut 'hassle and time factors'. Complex behavioural goals were simplified by breaking down complex goals into achievable steps and including supporting resources. *Attractive*: NHS branding was used to improve trustworthiness (11, 12) alongside infographics, personalisation and plain English wording. Health benefits of behaviour change were presented with alternative incentives to boost motivation to change. *Social*: Social norms messaging was used to motivate behaviour change and increase salience. The action planning section includes family and friends in goal setting, prompts discussion and encourages making a commitment to others. *Timely*: delivery of messages is optimal when in the appointment setting, but continues as patients reflect on and refer back to the report. Messaging highlights immediate as well as long term benefits of making behavioural changes.

The Risk Report includes QRISK2(13) and Heart Age (14) metrics to convey CVD risk, using infographics, icon arrays and pictorial natural frequencies with a common denominator to ease understanding and reduce denominator neglect.(15-18) Risk messages include a temporal component to provide context.(19) An infographic comparing comparative 'dread' risks is included to help patients situate their CVD risk alongside other causes of mortality.(20) We used survival framing to encourage risk-averse choices in terms of medication taking (21) and positive framing of messages to highlight alternative benefits of making healthy behaviours.(22) The Risk Report is printed in greyscale on four sides of A4 and includes information on local lifestyle change support services. It is available to view in full in the supplementary file.

Study design

Nested qualitative interview study within a randomised feasibility trial. A completed COREQ checklist for this study is available in the supplementary file.

Recruitment and selection

People aged 40-64 years due to be invited for an NHS Health Check were identified from six general practices in Newham, East London were invited to attend two checks, 3-6 months apart. A list of 250 patients were randomly allocated to intervention or control groups. The study code denoting assignment group was then entered in the GP record to identify these patients to the HCPs. The intervention group received a printed NHS Health Check risk report with a verbal explanation of its contents at the first check, and the control group received usual care without written advice at the first check and the risk report with a verbal explanation of its contents at the second check, following a waiting list control method. Patients in either group were eligible for the qualitative study after they had undertaken the two checks and received the risk report in either the first or second check.

Participants were recruited from three practices, out of six in the wider feasibility trial. Study information, consent form and an invitation to attend the NHS Health Check were provided in advance of recruitment. Participants gave written informed consent to take part in a qualitative

For peer review only - http://bmjopen.bmj.com/site/about/guidelines.xhtml

interview at enrolment into the feasibility trial, and again verbally prior to interview commencement.

Participants were purposively sampled according to the information held in the electronic health record following a maximum diversity sampling approach, according to four categories. These were: age, gender, ethnic group and CVD risk score (QRISK2(13)). QRISK2 score was categorised as low (a score of 10 or less), medium (score: 10-19) or high risk (score of 20 or more) according to the cut offs used by the NHS Health Check programme. Ethnicity as reported within the medical record were grouped into the following categories: white (British, European), black (Black (British/ African or Caribbean), South Asian (Indian, Pakistani, Bangladeshi, Sri Lankan), and all other ethnicities. Patients were telephoned and invited to participate by the project administrator or a practice receptionist.

Data collection

The development of the topic guide (box 1) was based on a literature review and was piloted at one of the practices. The topic guide used open questions and probes to gather more detail, and was adapted as new themes emerged. Single instance semi-structured interviews were undertaken in English, face to face in a private room at the practice by an experienced female qualitative researcher (MKDH) with a background in public health and health communication. Participants had no contact with the researcher before interview, and MKDH was introduced as a university researcher interested in improving NHS Health Checks. The Risk Report was introduced part way through the interview as a prompt and prop for discussion. Sessions lasted 10-40 minutes and were audio recorded, pseudonymised and then transcribed. Reflective field notes were taken after each interview. Data collection occurred March 2016 – July 2017, as part of the wider trial which took place March 2016 – December 2017. Interviews commenced until saturation occurred and no new themes were arising.(23) Interviewees received a £20 shopping voucher for their time.

Box 1: Topic Guide

- Early impressions of the NHS Health Check programme
- Motivations to attend/ accessibility
- Overall experience
- Key information/ messages
- Nurse/ HCA communication
- Understanding risk

--Introduce Risk Report--

- General feedback (first impressions/ comprehensiveness etc.)
- Design
- Suggestions for improvement
- Risk information
- Lifestyle changes
- Further comments

For peer review only - http://bmjopen.bmj.com/site/about/guidelines.xhtml

Analysis

Inductive thematic analysis of the qualitative data was undertaken,(24) which involved coding the transcripts according to latent and manifest content and developing a thematic framework.(24) Analysis by MKDH proceeded alongside data collection and NVIVO 10 software was used for data management.(25) 10% of transcripts were double coded by a second experienced qualitative researcher (JP). Attention was paid to the development of narratives within and across transcripts. Any inconsistencies were discussed and agreed amongst the research team to offer multiple perspectives on the development of codes and identification of themes, and overarching thematic connections.

Results

Sample Characteristics

18 respondents were well represented across gender, age group and self-defined ethnic group [Table 1]. Respondents at lower cardiovascular risk (QRISK2 score) were well represented but those at high risk were not represented in the sample. Of those approached, none refused to take part in the interview.

Table 1: Characteristics of sample

Group	Gender		Age Group (years)			QRISK2 score (%)			Ethnicity by grouping			
	Male	Female	40-50	51-60	60-74	10 or less	10 to 19	20 or more	White (British, European)	Black (British/ African or Caribbean)	South Asian (Indian, Pakistani, Bangladeshi, Sri Lankan)	All other Ethnicities
Control	3	4	2	4	1	6	1	0	0	3	2	2
Intervention	8	3	2	5	4	5	6	0	2	5	4	0
**Totals**	**11**	**7**	**4**	**9**	**5**	**11**	**7**	**0**	**2**	**8**	**6**	**2**

Overview of themes

Our thematic framework resulted in the identification of four main themes, which are outlined in Table 2. On further interpretation we recognised two overarching threads in our data. These were patterns in the linking of themes. We discuss the findings within each theme and sub-theme before discussing how the themes link together.

Table 2: Thematic framework

Differing Motivations	Multiple risks	Risk Report as an enduring record	Impact
	Binary risk<br>Risk meanings		
Detecting disease	Acceptance of risk	'Proof' of results	Making changes
Being a willing patient	Rejection of risk	Ambivalency	Not making changes

For peer review only - http://bmjopen.bmj.com/site/about/guidelines.xhtml

For peer review only

1. Differing Motivations

Detecting disease

Most participant accounts of why they attended the NHS Health Check were underscored by a desire to check their fitness, or to screen for previously undetected disease, especially in the context of increasing age. This was framed as 'maintaining your health'.

> *"C101: Because I, I'm over 50 now, so I guess now is the right time to say you will have a check on your health, and try and see how fit you are."*

There was a sense that some interviewees wanted to be reassured 'just in case', with reported fears linked to having a family history of CVD or previous test results, such as high blood pressure results.

Being a willing patient

Amongst a minority of interviewees, the reason they attended was out of a sense of obligation to the practice and to the NHS more generally. One participant wanted to be seen as a 'willing patient'. Another attended to make sure they did not get removed from the practice register.

> *"C103: Probably to keep in with the practice, you know, showing that I was a good, energetic, willing patient."*

2. Multiple risks

There were multiple constructions of 'risk' in the interviews, reflecting the differences in participants' perceptions of their own health, whether they were concerned and whether or not there were language barriers during the health check.

Binary risk

Those for whom English was not a first language mostly communicated their CVD risk in general, binary terms when asked, reporting that they were 'fine' or 'not at risk'. Risk for these interviewees was either a state of being 'at risk', or not. Percentage risk and/or heart age were not mentioned or discussed.

Risk meanings

Those that discussed risk in terms of their risk scores applied different meanings and importance to QRISK2 and heart age. For instance, this participant was explaining how she would like to have known her QRISK2 score, because the two types of risk have different meanings for her:

> *"C202: This [heart age] is good to look at, it makes me feel young and things like, oh yeah I'm healthy-ish but our heart age doesn't, it's nice but I would have liked to have known my [Q] risk, e… I think it would make you make more changes when you can actually see, OK, so I'm really at risk here […] because even though it's 44 yeah, I don't know the risks."*

For peer review only - http://bmjopen.bmj.com/site/about/guidelines.xhtml

Most participants were quicker to remember and report their heart age spontaneously in comparison to their QRISK2 score.

Acceptance of risk

Interviewees reacted to their CVD risk scores in varied ways based on their perceptions of their state of health before attending the check. Those who mentioned being concerned about their CVD health prior to attending the NHS Health Check were more likely to report their risk score and accept it as a 'true' reflection of their current state of health:

> *"C204: And she told me about the age, like my heart is 54 years old when I'm just 51. Yeah, you don't reach, I wanted it to be lower [...] I'm going to try my best to bring it lower. That's what I'm trying to do right now."*

Rejection of risk

Some participants already believed themselves to lead a healthy life or have a healthy heart and this meant that they disagreed with or discounted the CVD risk that was presented to them in the check if it did not fall in line with their own perception. For them, their risk was pre-defined by how they felt and saw themselves prior to the check:

> *"C103: Yeah, now that was a bit odd actually because the first time she said that there was a 1 in 10 chance of me having a heart attack and I thought, well that just doesn't seem right, once I got home I thought, no, that really doesn't seem right [...] So that was a bit odd but I thought, I'm not going to have one [a CVD event] anyway. No. [laughs]"*

For these participants, their perception of not being 'at risk' was based on feeling healthy and leading a healthy life, a lack of symptoms or the belief that good health is bestowed by a higher power.

3. Risk Report as an enduring record

The Risk Report prompted participants to recall their CVD risk results. First impressions of the risk report were positive, as this example shows:

"C110: I thought this was quite interesting, straightaway got the message across, the risk levels and things."

Participants found it to be 'user friendly' and clear with a good size font, no jargon and good for people for whom English was not their first language, or for those without a 'scientific' background.

*"C109: I'd just assume if my mother-in-Law came and did this, this would be just right for her. Whereas, yeah if, if there was another person with a scientific background, we might suggest even more [information] but yeah, I think that was enough."***'Proof' of results**

The majority of participants talked positively about the Risk Report as an enduring record of their results:

> *"C202: I think I liked the bit of paper that they gave me at the end that just jots down everything because I think you forget really easily and that's been good to look back at."*

For peer review only - http://bmjopen.bmj.com/site/about/guidelines.xhtml

For some, this had additional meaning, as 'proof' and reassurance of their good health, as this quote describes:

> *"C109: Having something positive as proof on a piece of paper, like you could actually, a physical piece of paper. I know it sounds silly right but having a, it's like, hey if the pain is still there, like I say, at least my heart's working properly."*

Participants reported looking back at the Risk Report to jog their memory. Some shared and discussed it with family and friends. Two reported keeping it by their bed, and some discussed how they would keep it to compare their results in future checks.

> *"C207: Some leaflets you can look at and throw it away but when you look at that box and you're look at it and you're thinking, oh yeah that's interesting. […] Yeah, it does motivate you, as it has for me anyway, it really has."*

Ambivalency

A few interviewees were not interested in the Risk Report as a record at all, had not looked back at it or engaged with it:

> *"I: Did you get a leaflet [showing Risk Report] like that?*
>
> *C205: Might be. I haven't opened it since. It might still be in the house."*

4. Impact

Making changes

Most patients reported making at least small changes as a result of the NHS Health Check, such as incorporating more walks, reducing their salt and fat intake, and cutting down on alcohol consumption. A few participants had made significant changes – for instance one patient had begun exercising regularly, joined a gym with his partner, cut down on fried foods, avoided drinking strong spirits and begun smoking less, reporting his cholesterol reduced from 5.5 to 4.9 in three months.

Not making changes

There were a few patients who had not made any changes to their lifestyle after the Check. These tended to be patients who reported that they were low risk or who already felt they were in good health. One patient, who had a medium QRISK2 score, was pre-diabetic and had been referred on for further tests, had tried to make changes but did not manage to keep it up:

> *"C104: Well I want to but you've not got the go all the time, sometimes you just want to binge, what I do a lot. […] The diet plans, my son printed some sheets off from work, what you're supposed to be eating, but you'd do it for a few days and then you put them in a drawer."*

Another patient had also been referred on for more tests to investigate his glucose levels but had not attended the referral clinic. He clearly understood his risk and what had been asked of him, however did not change his behavior according to the NHS Health Check results and recommendations:

For peer review only - http://bmjopen.bmj.com/site/about/guidelines.xhtml

> *"C205: I was supposed to go for that check up […] And I didn't go for that. […] As long as I can still run and walk and I don't have pains and aches and dizzy spells as they like to call them. I don't. I don't worry."*

Wider impact

It was clear from the interviews that there was a wider social impact of NHS Health Checks that went beyond individuals. This included sharing the information from the Check with others, family members being included in diet and exercise changes, as well as recommendations to family and friends that they should attend their NHS Health Check.

> *"C202: So now I have to look at my diet and actually even for my children as well, and look at their diet and just think, is it necessary, do we need all this salt, and I do really like salt and we are like a salty family. I would just add salt to everything and now I'm starting to think that I can't."*

> *"C207: I showed her [wife] and yeah and I've looked at it and it's when we go shopping now we, we probably have more vegetables and fruits in our shopping trolley than we have of all crisps."*

5. Bringing the themes together

The majority of participants' accounts played out according to the main thematic findings described above. Participants attended their NHS Health Check to assess their levels of fitness and to get reassurance that nothing was wrong, or because they were concerned about previous tests or predisposition to ill-health. They accepted the level of risk that the screening assigned to them, and viewed the Risk Report as a positive enduring record of their health status. They then went on to make lifestyle changes and share these with their families.

A minority of participant accounts in the study followed a different narrative from the central themes. These participants attended out of obligation or duty rather than concern for their health. They did not readily accept or internalise risk scores that differed from their own assessments of being healthy. They were ambivalent towards the Risk Report and did not make advised lifestyle changes. These diverse cases were few but are important for our consideration and recognition.

Discussion

Summary

This study sought to explore patient experiences of a personalised Risk Report, designed to improve cardiovascular risk communication in the NHS Health Check. For most, the NHS Health Check was an opportunity for reassurance and assessment, and the Risk Report was an enduring record that supported risk understanding, and supported lifestyle change for the individual and their wider social networks. For a minority, ambivalence towards the Risk Report occurred in the context of attending for other reasons, and risk and lifestyle advice were not internalised or acted upon.

Strengths and Limitations

For peer review only - http://bmjopen.bmj.com/site/about/guidelines.xhtml

Our sample included medium and low CVD risk participants from a range of ethnic groups and ages, reflecting the Newham population, of whom the majority are low to medium risk.(9) Due to the targeted delivery approach adopted in the local area and high uptake rates in this area,(9) at the time of the study a large proportion of high risk patients had already been identified and referred for further specialist services and support. In contrast, many low to medium risk patients are unlikely to receive further clinical referrals as a result of the NHS Health Check, and as such, the Risk Report may be the only intervention that they receive. This group are therefore most likely to benefit from the action planning and further resources sections provided within the risk report, and are an appropriate target group for this intervention, and as the focus of this study. The majority (16/18) of participants in this study were from black and ethnic minority (BME) backgrounds, which reflects in part the diversity of the population in Newham Borough. Whilst this is an encouraging start, further adaptation may be required to tailor particular elements of the risk report for diverse population groups and cultural backgrounds.

Our findings may not be transferrable to those who do not take up the offer of the NHS Health Check, nor those who do not speak English. Participants were recruited from a wider feasibility trial, and so were patients who had both consented to the check and to take part in research. Participants were from both control and intervention groups, but, as both received the risk report either at the first check or the second (3 months apart), and patients were not aware which group they were allocated to, this may not have had a big impact on the data we collected. The inclusion of four patients for whom English was not their first language was pragmatic and reflects routine care in many areas, where health care professionals must communicate with patients about CVD risk regardless of language barriers and without the assistance of professional translators. Our finding that risk is constructed as 'binary' as a result of language barriers, even in the presence of a risk report with explanatory graphics, shows how risk is constructed in a fundamentally different way in comparison to numerical constructions of risk used by patients without those communication barriers. The impact this has on perceptions of health and health outcomes amongst this group warrants further investigation. Specific adaptations to the Risk Report and the NHS Health Check itself are required to extend the programme more equitably to all types of patient groups.

Comparison with Existing literature

Familial experience with CVD (26) and concerns about preventable or undetected illness as motivators to attend (27, 28) have been reported elsewhere. Some participants in our study presented due to specific concerns, such as high blood pressure, supporting Perry's assertion that it is not only the 'worried well' who attend.(4) Burgess and colleagues (29) found civic responsibility to be a driver for some patients, which falls in line with being seen as a 'willing patient'. This study builds on these findings by aligning motivations to attend with the ongoing experiences of having a health check.

One key finding was the notion of CVD risk as a binary category, rather than a percentage scale. The 'take home message' from the NHS Health Check was often limited to dualistic generalities like being 'fine' or 'not at risk'. Whether people at higher CVD risk receive meaningful information and people at lower CVD risk are not falsely reassured should be a key focus of research in this area. The concept of an "MOT", or 'roadworthiness'(30) - used prominently in the advertising of some NHS Checks - in which some people 'pass' – and presumably need take no further action - and the others 'fail' may not be the most effective message to convey. van Steenkiste and colleagues (31) found

For peer review only - http://bmjopen.bmj.com/site/about/guidelines.xhtml

that communicated test results faded into an 'overall reassuring message' instead of specific results. Numerically, most heart attacks and strokes occur in people at moderate CVD risk, so if health checks are to be successful the focus must remain on the importance of reducing the burden of CVD in all patients, not just those at high risk. Challenging the 'MOT' metaphor associated with the overall programme may also help in this regard.

A key finding of our study was the ways in which patients used the Risk Report to not only motivate their own lifestyle changes, but as a way to prompt and support discussions and changes with others in their family and broader social networks. This finding has been mirrored in ethnographic work in a community health check setting, where Afro-Caribbean participants went on to discuss their results with peers.(26) In our study the 'work' of becoming healthier was a shared endeavor, involving those around the 'patient', including older relatives and children. Information impacted beyond the person through social practices such as eating and being physically active. Moving away from an individualistic approach to examine shared practices in the aftermath of health checks may be a fruitful area for future research.

The majority of participant views aligned with the dominant biomedical risk narrative behind screening for preventable illness in the UK. This viewpoint places the responsibility for maintaining health to the individual, via 'correct' lifestyle choices.(32) Having an NHS Health Check feeds this narrative by offering the opportunity for reassurance and provision of guidance to make small changes to maintain or protect health from uncertainty, or risk. However, we found a minority of participants did not engage with the risk element of the NHS Health Checks, or intend to make lifestyle changes as a result of having a check, instead they attended for other reasons and were ambivalent about the risk report and the idea of 'improving' their health. These patients are often characterised as 'non-attenders' without an attempt to explore their worldview.(33, 34) Models of continuous care, typified by trusting clinician-patient relationships (35) and attention to patient narratives (36) may allow for better ways of communicating about cardiovascular ill-health with patients whose perspectives do not align with the dominant biomedical narrative. Additionally, qualitative attention to the whole patient and their journey through the NHS Health Check and beyond could help identify alternative ways of delivering good care for these groups of patients.

Implications for research and practice

Our qualitative results support further development of a Risk Report for NHS Health Checks, to enhance risk communication and support lifestyle modification and dissemination of messages amongst wider social networks. Our findings also highlight some of the challenges faced by the NHS Health Check programme for supporting patients whose ideas about risk and screening for future illness don't align with NHS and preventative public health priorities. Shifting the focus of the NHS Health Checks beyond the individual, to consider how networks of family and friends might interact, may have positive influences for leading healthier lives.

Additional information

For peer review only - http://bmjopen.bmj.com/site/about/guidelines.xhtml

Funding

This study was funded by The Guttmann Academic Partnership hosted by UCLPartners. Meredith K.D. Hawking was in part supported by the National Institute for Health Research (NIHR) Collaboration for Leadership in Applied Health Research and Care North Thames at Bart's Health NHS Trust (NIHR CLAHRC North Thames). The views expressed in this article are those of the author(s) and not necessarily those of the NHS, the NIHR, or the Department of Health and Social Care.

Ethical Approval

The study received final approval from the National Research Ethics Service Committee North West (Preston) on 7/8/2015. The research ethics committee reference was: 15/NW/0635 (protocol number MCPH1C8R). The study was registered with ClinicalTrials.gov (NCT02486913).

Competing Interests

MKDH, JLP, AT and FW have no interests to declare. JR has a non-pecuniary interest as a co-author of QRISK2 and is also a member of a national NHS Health Check advisory group.

Acknowledgements

We are grateful to the NHS Health Check attendees for giving up their time to take part in the study and share their experiences. We also extend our thanks for the cooperation and support from general practitioners and the clinic staff who were involved in delivering the study and those who helped inform the development of the Risk Report.

Contributorship

MKDH led the design of the risk report and with AT and JR designed the qualitative study. FW organised the recruitment of practices, IT components, staff training and participant attendance. MKDH carried out the interviews and analysed the transcripts with JLP. All authors contributed to the manuscript. Patients/ the public were not directly involved in the research process.

Data sharing statement

No data are available. We did not seek ethical approval or patient consent for the full transcripts to be shared beyond the research team.

References

1. DOH. Putting Prevention First – NHS Health Check: Vascular Risk Assessment and Management Best Practice Guidance. London: Department of Health; 2009.
2. Baker C, Loughren EA, Crone D, Kallfa N. Patients' perceptions of a NHS Health Check in the primary care setting. Quality in primary care. 2014;22(5):232-7.
3. Riley R, Coghill N, Montgomery A, Feder G, Horwood J. Experiences of patients and healthcare professionals of NHS cardiovascular health checks: a qualitative study. Journal of public health (Oxford, England). 2016;38(3):543-51.

For peer review only - http://bmjopen.bmj.com/site/about/guidelines.xhtml

4. Perry C, Thurston M, Alford S, Cushing J, Panter L. The NHS health check programme in England: a qualitative study. Health promotion international. 2016;31(1):106-15.
5. Krska J, du Plessis R, Chellaswamy H. Views and experiences of the NHS Health Check provided by general medical practices: cross-sectional survey in high-risk patients. Journal of Public Health. 2015;37(2):210-7.
6. McNaughton RJ, Shucksmith J. Reasons for (non)compliance with intervention following identification of 'high-risk' status in the NHS Health Check programme. Journal of public health (Oxford, England). 2015;37(2):218-25.
7. Ismail H, Atkin K. The NHS Health Check programme: insights from a qualitative study of patients. Health Expect. 2016;19(2):345-55.
8. Shaw RL, Lowe H, Holland C, Pattison H, Cooke R. GPs' perspectives on managing the NHS Health Check in primary care: a qualitative evaluation of implementation in one area of England. BMJ open. 2016;6(7):e010951.
9. Robson J, Dostal I, Madurasinghe V, Sheikh A, Hull S, Boomla K, et al. The NHS Health Check programme: implementation in east London 2009–2011. BMJ open. 2015;5(4).
10. Service OH, M.; Halpern, D.; Algate, F.; Gallagher, R.; Nguyen, S.; Ruda, S.; Sanders, M.; Pelenur, M.; Gyani, A.; Harper, H.; Reinhard, J.; Kirkman, E. EAST: Four ways to apply behavioural insights. London: Behavioural Insights Ltd; 2014.
11. Powell J, Inglis N, Ronnie J, Large S. The Characteristics and Motivations of Online Health Information Seekers: Cross-Sectional Survey and Qualitative Interview Study. J Med Internet Res. 2011;13(1):e20.
12. Quiroz-Aitken M, Cunningham-Burley S, Pagliari C. Moving from Trust to Trustworthiness: Experiences of public engagement in the Scottish Health Informatics Programme. Science and Public Policy. 2016.
13. Hippisley-Cox J, Coupland C, Vinogradova Y, Robson J, Minhas R, Sheikh A, et al. Predicting cardiovascular risk in England and Wales: prospective derivation and validation of QRISK2. BMJ. 2008;336(7659):1475.
14. JBS3. Joint British Societies' consensus recommendations for the prevention of cardiovascular disease (JBS3). Heart. 2014;100(Suppl 2):ii1.
15. Kurz-Milcke E, Gigerenzer G, Martignon L. Transparency in risk communication: graphical and analog tools. Annals of the New York Academy of Sciences. 2008;1128:18-28.
16. Goodyear-Smith F, Arroll B, Chan L, Jackson R, Wells S, Kenealy T. Patients prefer pictures to numbers to express cardiovascular benefit from treatment. Annals of family medicine. 2008;6(3):213-7.
17. Garcia-Retamero R, Galesic M, Gigerenzer G. Do icon arrays help reduce denominator neglect? Med Decis Making. 2010;30(6):672-84.
18. Galesic M, Garcia-Retamero R, Gigerenzer G. Using icon arrays to communicate medical risks: overcoming low numeracy. Health psychology : official journal of the Division of Health Psychology, American Psychological Association. 2009;28(2):210-6.
19. Waldron CA, van der Weijden T, Ludt S, Gallacher J, Elwyn G. What are effective strategies to communicate cardiovascular risk information to patients? A systematic review. Patient Educ Couns. 2011;82(2):169-81.
20. Gigerenzer G. Dread risk, September 11, and fatal traffic accidents. Psychological science. 2004;15(4):286-7.
21. Veldwijk J, Essers BAB, Lambooij MS, Dirksen CD, Smit HA, de Wit GA. Survival or Mortality: Does Risk Attribute Framing Influence Decision-Making Behavior in a Discrete Choice Experiment? Value in Health. 2016;19(2):202-9.
22. Akl EA, Oxman AD, Herrin J, Vist GE, Terrenato I, Sperati F, et al. Framing of health information messages. The Cochrane database of systematic reviews. 2011(12):Cd006777.
23. Mason M. Sample Size and Saturation in PhD Studies Using Qualitative Interviews. 2010. 2010;11(3).

For peer review only - http://bmjopen.bmj.com/site/about/guidelines.xhtml

24. Braun V, Clarke V. Using thematic analysis in psychology. Qualitative Research in Psychology. 2006;3(2):77-101.
25. QSR, International, Pty, Ltd. NVivo Qualitative Data Analysis Software Version 10. 2012.
26. Riley R, Coghill N, Montgomery A, Feder G, Horwood J. The provision of NHS health checks in a community setting: an ethnographic account. BMC health services research. 2015;15:546.
27. Ellis N, Gidlow C, Cowap L, Randall J, Iqbal Z, Kumar J. A qualitative investigation of non-response in NHS health checks. Archives of Public Health. 2015;73(1):1-8.
28. Jenkinson CE, Asprey A, Clark CE, Richards SH. Patients' willingness to attend the NHS cardiovascular health checks in primary care: a qualitative interview study. BMC Fam Pract. 2015;16:33.
29. Burgess C, Wright AJ, Forster AS, Dodhia H, Miller J, Fuller F, et al. Influences on individuals' decisions to take up the offer of a health check: a qualitative study. Health Expect. 2015;18(6):2437-48.
30. Shaw RL, Pattison HM, Holland C, Cooke R. Be SMART: examining the experience of implementing the NHS Health Check in UK primary care. BMC Fam Pract. 2015;16:1.
31. van Steenkiste B, van der Weijden T, Timmermans D, Vaes J, Stoffers J, Grol R. Patients' ideas, fears and expectations of their coronary risk: barriers for primary prevention. Patient Education and Counseling. 2004;55(2):301-7.
32. Lupton D. Risk as Moral Danger: The Social and Political Functions of Risk Discourse in Public Health. International Journal of Health Services. 1993;23(3):425-35.
33. Dalton A, Bottle R, Okoro C, Majeed F, Millett C. Uptake of the NHS health checks programme in a deprived, culturally diverse setting: cross sectional study. J Epidemiol Commun H. 2011;65.
34. Dryden R, Williams B, McCowan C, Themessl-Huber M. What do we know about who does and does not attend general health checks? findings from a narrative scoping review. BMC Public Health. 2012;12.
35. Robb N, Greenhalgh T. "You have to cover up the words of the doctor": The mediation of trust in interpreted consultations in primary care. Journal of Health Organization and Management. 2006;20(5):434-55.
36. Greenhalgh T, Collard A, Campbell-Richards D, Vijayaraghavan S, Malik F, Morris J, et al. Storylines of Self-Management: Narratives of People with Diabetes from a Multiethnic Inner City Population. Journal of Health Services Research & Policy. 2011;16(1):37-43.

For peer review only

For peer review only - http://bmjopen.bmj.com/site/about/guidelines.xhtml

List of figures

Figure 1: Risk Report

Box 1: Topic Guide

Table 1: Characteristics of sample

Table 2: Thematic framework

For peer review only

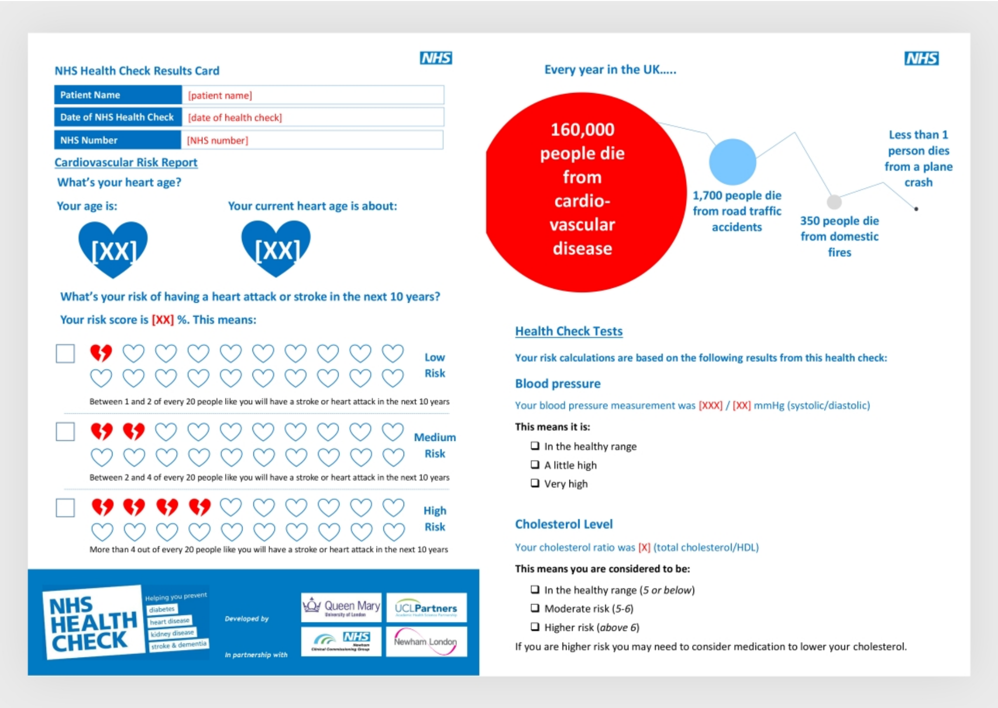

Figure 1: Risk Report

297x209mm (300 x 300 DPI)

NHS Health Check Results Card

**Patient Name**	[patient name]
**Date of NHS Health Check**	[date of health check]
**NHS Number**	[NHS number]

Cardiovascular Risk Report

What's your heart age?

Your age is:

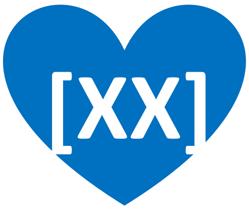

Your current heart age is about:

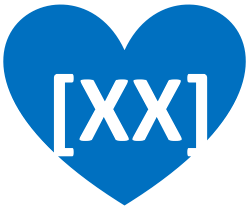

What's your risk of having a heart attack or stroke in the next 10 years?

Your risk score is [XX] %. This means:

☐ 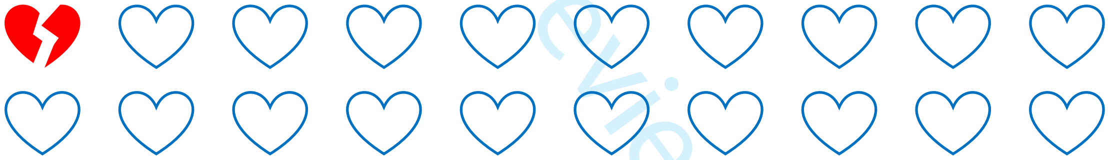

Low Risk

Between 1 and 2 of every 20 people like you will have a stroke or heart attack in the next 10 years

☐ 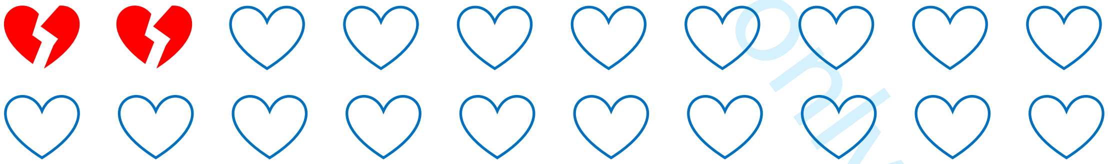

Medium Risk

Between 2 and 4 of every 20 people like you will have a stroke or heart attack in the next 10 years

☐ 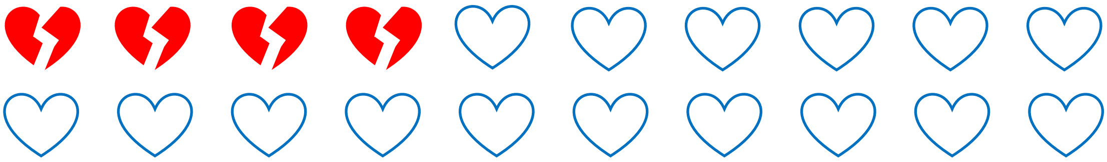

High Risk

More than 4 out of every 20 people like you will have a stroke or heart attack in the next 10 years

NHS HEALTH CHECK
Helping you prevent
diabetes
heart disease
kidney disease
stroke & dementia

Developed by

Queen Mary University of London
UCLPartners Academic Health Science Partnership

In partnership with

NHS Newham Clinical Commissioning Group
Newham London

For peer review only

Every year in the UK.....

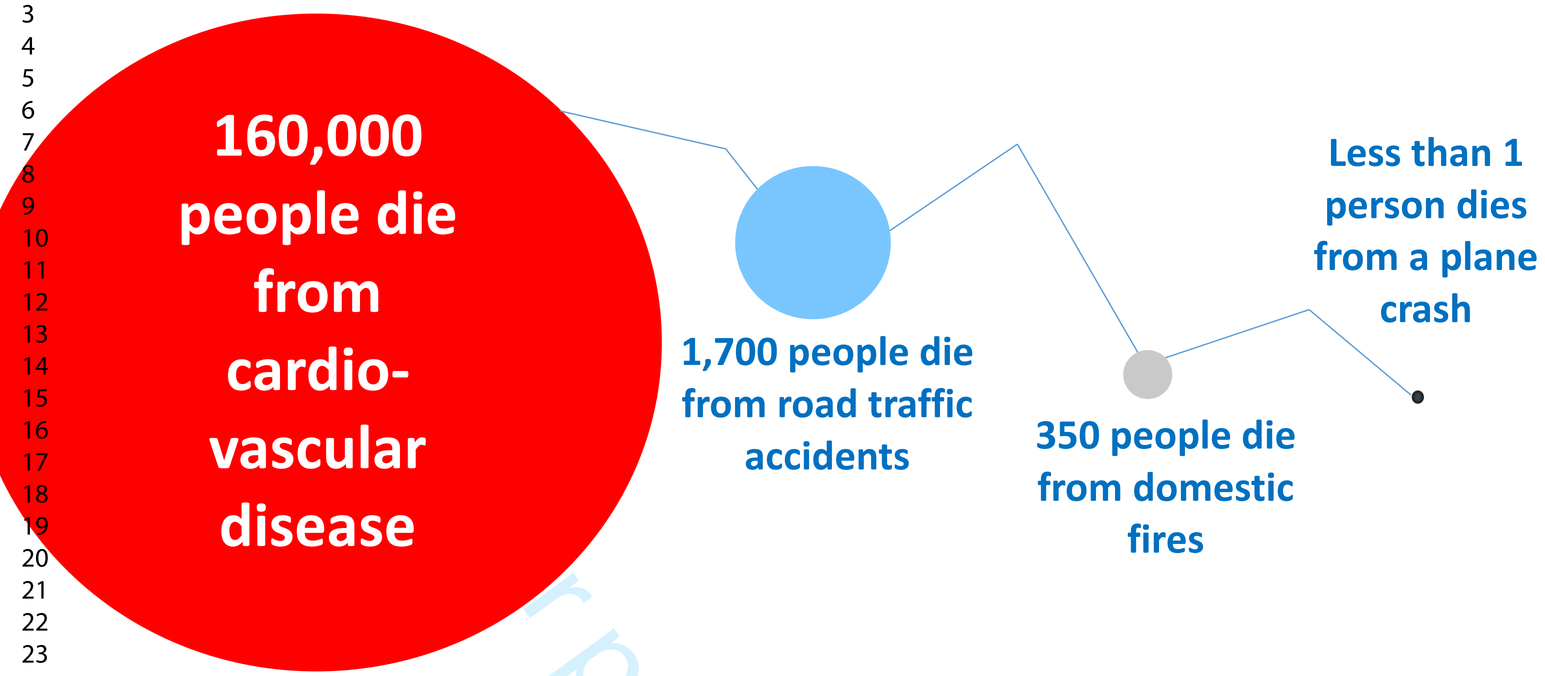

Health Check Tests

Your risk calculations are based on the following results from this health check:

Blood pressure

Your blood pressure measurement was [XXX] / [XX] mmHg (systolic/diastolic)

This means it is:

- ❑ In the healthy range
- ❑ A little high
- ❑ Very high

Cholesterol Level

Your cholesterol ratio was [X] (total cholesterol/HDL)

This means you are considered to be:

- ❑ In the healthy range (*5 or below*)
- ❑ Moderate risk (*5-6*)
- ❑ Higher risk (*above 6*)

If you are higher risk you may need to consider medication to lower your cholesterol.

For peer review only

Diabetes Check

Your glycated haemoglobin (HbA1c) result was [X.X] %

This means that you:

- ❑ Do not have diabetes (*less than 42*)
- ❑ Are at risk of developing diabetes (*42-47*)
- ❑ May have diabetes and will need to have further testing (*48 or over*)

Body Mass Index

Your BMI was [XX]

This means that you are:

White, Black African/Caribbean or other ethnic groups:

- ❑ Healthy weight (*18.5 to less than 25*)
- ❑ Overweight (*25 to less than 30*)
- ❑ Obesity 1 (*30 to less than 35*)
- ❑ Obesity 2 (*35 to less than 40*)
- ❑ Obesity 3 (*40 or more*)

South Asian or other Asian:

- ❑ Healthy weight (*18.5 to less than 23*)
- ❑ Overweight (*23 to less than 27.5*)
- ❑ Obesity 1 (*27.5 to less than 32.5*)
- ❑ Obesity 2 (*32.5 to less than 37.5*)
- ❑ Obesity 3 (*37.5 or more*)

For peer review only

You said you smoke [XX] cigarettes a day

66% of smokers would like to give up the habit. Giving up 20 premium brand cigarettes a day would save approximately £3,000 a year. You are up to four times more likely to quit if you use NHS support than if you go it alone.

All areas have a free local NHS Stop Smoking Service which can help you find your best way of stopping, providing the medication and support you need. Call 0300 123 1044 for more information.

You said you drink [XX] units of alcohol per week

Cutting out a couple of large glasses of wine or beer each week could save you more than £400 year, as well as helping you to lose weight and get better quality sleep.

Drinking within recommended guidelines (no more than 14 units per week for men and women), not regularly drinking more 2-3 units a day, and having at least two alcohol free days per week will reduce your risk.

NHS

Your Personal Action Plan

Now that you know your cardiovascular risk and heart age, what can you do to improve it?

- ❑ Lose some weight

  Sign up to a weight loss programme, cut out foods and drinks with high levels of sugar and fats

- ❑ Eat more healthily

  Eat your 5 a day, swap out sugary drinks for diet or sugar-free versions, choose wholegrain foods such as wholemeal bread and pasta and brown rice, cook using fresh ingredients at home

- ❑ Do more exercise

  Sign up to a sports group or gym, take the stairs instead of lifts, walk and cycle to work

- ❑ Reduce your blood pressure and cholesterol

  Grill, bake, steam and poach meat instead of frying or roasting, eat more fibre (wholegrains such as wholemeal bread and pasta and vegetables), eat less fatty meats, dairy foods, ghee and cakes and biscuits

- ❑ Reduce your salt intake

  Add less salt to your cooking or plate, check labels on ready-made foods

- ❑ Stop smoking

  Order your Smokefree quit kit today, save your cigarette money in a pot to see how it builds up

- ❑ Reduce the amount of alcohol you drink

  Use smaller glasses or measures when pouring wine and spirits at home, have a few drink-free days each week, alternate alcoholic drinks with glasses of water, drink with a meal

For peer review only

What goal(s) would you like to set yourself?

What small changes would help you achieve your goal(s)?

When will you achieve this?

Who can help you achieve this goal?

For more information and support about how to achieve these goals, visit or call:

- NHS Health Check w: www.nhs.uk/Conditions/nhs-health-check
- NHS free weight loss plan w: www.nhs.uk/Livewell/weight-loss-guide
- Change for life w: www.nhs.uk/change4life t: 0300 123 4567
- Smokefree NHS w: www.nhs.uk/smokefree t: 0300 123 1044
- NHS Alcohol information w: www.nhs.uk/Livewell/alcohol
- Action on Salt w: www.actiononsalt.org.uk t: 020 7882 5941
- Diabetes w: www.diabetes.co.uk
- Dementia w: www.alzheimers.org.uk t: 0300 222 1122

What's good for the heart is good for the head

Treatment:

After this health check, you may need a follow up appointment, and your doctor may prescribe you some medication to manage your risk of cardiovascular disease. You will be told by the clinic staff if you need to make another appointment.

For every 20 potential strokes or heart attacks, treatment will prevent 7:

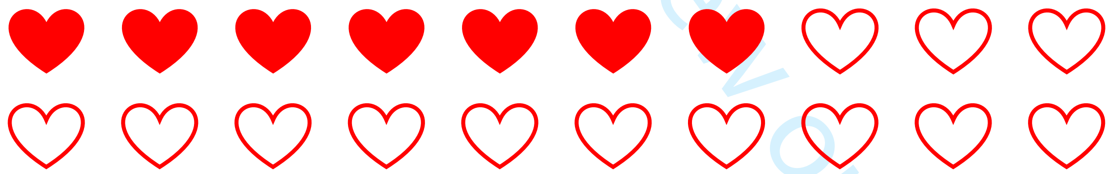

- [ ] Remember to take any prescribed treatment according to your doctor's instructions

Set the alarm on your phone to remind you or put the pills next to your toothbrush

Extra information/ Local Support Groups:

Your next appointment will be due in……………………………………………………………………………………..

This NHS Health Check Risk Report was devised by the Clinical Effectiveness Group QMUL in collaboration with the East London CVD Prevention team at Bart's Heart Centre. It is licensed under the Creative Commons Attribution-NonCommercial 4.0 International License. To view a copy of this license, visit http://creativecommons.org/licenses/by-nc/4.0/ or send a letter to Creative Commons, PO Box 1866, Mountain View, CA 94042, USA.

For peer review only - http://bmjopen.bmj.com/site/about/guidelines.xhtml

Table 1

Consolidated criteria for reporting qualitative studies (COREQ): 32-item checklist

**No**	**Item**	**Guide questions/description**		**Page**
**Domain 1: Research team and reflexivity**				
Personal Characteristics				
1.	Interviewer/facilitator	Which author/s conducted the interview or focus group?	MKDH	5
2.	Credentials	What were the researcher's credentials? *E.g. PhD, MD*	MPH, BSc	1
3.	Occupation	What was their occupation at the time of the study?	Research assistant	1
4.	Gender	Was the researcher male or female?	F	5
5.	Experience and training	What experience or training did the researcher have?	Training in qualitative research and previous experience in health related communication	5
Relationship with participants				

For peer review only

No	Item	Guide questions/description		Page
6.	Relationship established	Was a relationship established prior to study commencement?	No	5
7.	Participant knowledge of the interviewer	What did the participants know about the researcher? e.g. *personal goals, reasons for doing the research*	Participants were informed that MKDH was a university researcher conducting a study	5
8.	Interviewer characteristics	What characteristics were reported about the interviewer/facilitator? e.g. *Bias, assumptions, reasons and interests in the research topic*	Informed MKDH was interested in improving NHS Health Checks	5
**Domain 2: study design**				
Theoretical framework				
9.	Methodological orientation and Theory	What methodological orientation was stated to underpin the study? *e.g. grounded theory, discourse analysis, ethnography, phenomenology, content analysis*	Inductive thematic analysis with coding of transcripts according to latent and manifest content and developing a thematic framework.	6

For peer review only

No	Item	Guide questions/description		Page
Participant selection				
10.	Sampling	How were participants selected? e.g. *purposive, convenience, consecutive, snowball*	Purposive maximum diversity sample	5
11.	Method of approach	How were participants approached? e.g. *face-to-face, telephone, mail, email*	Mail and face to face	5
12.	Sample size	How many participants were in the study?	18	6
13.	Non-participation	How many people refused to participate or dropped out? Reasons?	None	6
Setting				
14.	Setting of data collection	Where was the data collected? e.g. *home, clinic, workplace*	General practice clinic	5
15.	Presence of non-participants	Was anyone else present besides the participants and researchers?	No one	5
16.	Description of sample	What are the important characteristics of the sample? *e.g. demographic data, date*	Demographic	7
Data collection				
17.	Interview guide	Were questions, prompts, guides	Pilot tested	5/6

For peer review only

No	Item	Guide questions/description		Page
		provided by the authors? Was it pilot tested?	The topic guide [box 1] was piloted, used open questions and probes to gather more detail, and was adapted as new themes emerged.	
18.	Repeat interviews	Were repeat interviews carried out? If yes, how many?	No	5
19.	Audio/visual recording	Did the research use audio or visual recording to collect the data?	Yes audio recording	5
20.	Field notes	Were field notes made during and/or after the interview or focus group?	Yes	5
21.	Duration	What was the duration of the interviews or focus group?	10-40 minutes	5
22.	Data saturation	Was data saturation discussed?	Yes	5
23.	Transcripts returned	Were transcripts returned to participants for comment and/or correction?	No	5
**Domain 3: analysis and findings**z				

For peer review only

No	Item	Guide questions/description		Page
Data analysis				
24.	Number of data coders	How many data coders coded the data?	2	6
25.	Description of the coding tree	Did authors provide a description of the coding tree?	No	6
26.	Derivation of themes	Were themes identified in advance or derived from the data?	Derived from data	6
27.	Software	What software, if applicable, was used to manage the data?	NVIVO 10	6
28.	Participant checking	Did participants provide feedback on the findings?	No	6
Reporting				
29.	Quotations presented	Were participant quotations presented to illustrate the themes / findings? Was each quotation identified? e.g. *participant number*	Yes; each participant is identified	7-11
30.	Data and findings consistent	Was there consistency between the data presented and the findings?	Yes	7-11
31.	Clarity of major themes	Were major themes clearly presented in the findings?	Yes	7

For peer review only

No	Item	Guide questions/description		Page
32.	Clarity of minor themes	Is there a description of diverse cases or discussion of minor themes?	Yes	11

For peer review only
